# On the Durability of Icephobic Coatings: A Review

**DOI:** 10.3390/ma17010235

**Published:** 2023-12-31

**Authors:** Andrés Nistal, Benjamín Sierra-Martín, Antonio Fernández-Barbero

**Affiliations:** Applied Physics, Department of Chemistry and Physics, University of Almeria, 04120 Almeria, Spain; bsierra@ual.es (B.S.-M.); afernand@ual.es (A.F.-B.)

**Keywords:** icephobic coating, anti-icing, deicing, durability

## Abstract

Ice formation and accumulation on surfaces has a negative impact in many different sectors and can even represent a potential danger. In this review, the latest advances and trends in icephobic coatings focusing on the importance of their durability are discussed, in an attempt to pave the roadmap from the lab to engineering applications. An icephobic material is expected to lower the ice adhesion strength, delay freezing time or temperature, promote the bouncing of a supercooled drop at subzero temperatures and/or reduce the ice accretion rate. To better understand what is more important for specific icing conditions, the different types of ice that can be formed in nature are summarized. Similarly, the alternative methods to evaluate the durability are reviewed, as this is key to properly selecting the method and parameters to ensure the coating is durable enough for a given application. Finally, the different types of icephobic surfaces available to date are considered, highlighting the strategies to enhance their durability, as this is the factor limiting the commercial applicability of icephobic coatings.

## 1. Introduction

Ice formation and accretion on surfaces, when not controlled or alleviated, are a great concern for many different industrial sectors, spanning from transport to clean energy or civil engineering. For instance, anti-icing/deicing (AI/DI) systems are of the utmost importance on aviation [1], as ice accreted on a wing increases drag and decreases lift [2] or can disturb the performance of key instruments such as the pilot tubes used to determine the airspeed [3]. Beyond the aerospace sector, other transport industries are also affected by icing events, such as the railway sector [4,5], where iced overhead catenaries can cause equipment malfunction and train delays. In the maritime sector [6,7,8] (where the offshore platforms can be also included), ice formation and accretion compromise the safety of marine operations in many different ways. Ice on the superstructure, deck, or walkways may cause accidents due to slippage or falling ice, or even capsize a vessel due to the loss of stability by lateral ice weight. Ice may jam mechanisms such as the anchor or block doors frozen shut. In the same way, ice accretion can drastically affect the performance of different systems related with the energy sector. Wind power systems’ efficiency can be reduced by ice accreted on the wind turbine blades [9,10]. Power line networks can be disrupted due to excessive weight on the overhead cables caused by ice accretion [11] and the sunbeams that reach solar cells can be scattered and reflected by an ice/snow layer [12], to name a few undesired scenarios. 

In order to prevent ice formation and accretion on a surface, a number of engineered solutions have been developed. The strategies to enhance the icephobic performance of a surface can be classified as single to a few icing event solutions, such as the application of salts for highways [13], glycols for aircrafts [14] or long term solutions such as icephobic coatings [7,15,16]. The application of chemicals is not only less effective but also implies concerns about its environmental impact [17], even though it is still the preferred solution in some specific cases due to its low cost. In order to determine the most suitable solution for a given application, several aspects are commonly considered. For example, the application of sodium chloride is by far the most widely used deicing strategy for road infrastructure due to its abundance and low cost [13], but it has important drawbacks to consider, such as the pollution of the soil and corrosion of the drainage systems and other infrastructures. Not in vain, asphalt concrete with improved icephobic performance is an active research area [18]. Likewise, there are efforts focused on the development of new AI/DI techniques for the aerospace sector, also triggered by the steady increment of carbon-fiber-reinforced polymers (CFRPs) replacing conventional aluminum-based aircrafts. The traditional solutions such as the application of freezing point depressants [17], the use of thermal-based deicers [5,19] or vibration/surface deformation-based systems [20,21,22,23] are usually combined with surfaces having a low ice adhesion strength (IAS) by using commercial icephobic coatings [24].

It is also very common to classify the icephobic approaches as active or passive, depending on the necessity of an external energy input or action. There are many automated mechanical methods, such as the use of pneumatic boots for aircrafts and wind turbines [25], the use of vibrational methods, either combined with ultrasonic guided waves [21] or with piezoelectric actuators [23,26], and even manual ones, such as the use of wooden baseball bats and similar tools, commonly used in offshore platforms and vessels operating in polar waters [27]. There are also thermal systems spanning from the heat pipes [28] traditionally used in aluminum-based aircrafts to electro-thermal systems [29] embedded in the laminated CFRP composites used in the last generation of aircrafts. According to this classification, passive/active approaches are considered AI/DI solutions, respectively. It is important to note that typical active deicing methods involve high energy consumption and the corresponding equipment is usually difficult to manufacture and requires considerable cost maintenance, and thus its use should be avoided or minimized as much as possible. On the contrary, a passive solution (icephobic surface) is always highly desired regardless of the presence/absence of an active deicing system, and in general terms, AI/DI solutions can be combined [30].

There are a countless number of applications where the ice formation and accretion should be avoided or at least minimized. In order to satisfy the requirements for a specific practical application, there might be other properties that an icephobic coating should meet, e.g., high transparency to UV/Vis light for solar cells or windows in the clean energy and transport sectors, respectively. To analyze the suitability of an icephobic coating for a specific application, the first aspect to consider will be the icephobic performance of the coating itself, and right after it, the longevity or durability, cost effectiveness and ease of application should be addressed, not necessarily in this order [31]. In addition, there are other aspects that might be important to consider, such as the environmental sustainability or even the aesthetics applications. 

Regardless of the application, the durability of an icephobic coating is a universal requirement given the fact that they are meant to remain effective under very harsh environments, usually outdoors, with very few specific exceptions such as freezers. In addition to the importance of the resilience of an icephobic coating on its long-term performance, there is a lack of consensus on how to measure and compare the durability of a coating. This review is intended to shed light on several important questions of icephobic coatings, bearing in mind the importance of their durability. The subject addressed starts with the primary basic question: what is an icephobic surface? The most common description of an icephobic surface is focused on its ability to prevent ice formation or facilitate its detachment. Thereafter, the following questions are tackled: How is ice formed and accreted? How can the icephobicity and durability of an icephobic surface be measured? How can the durability of different icephobic materials be enhanced? Finally, once these questions are discussed, some conclusions and prospects are drawn.

## 2. What Is an Icephobic Surface?

Icephobicity is generally ascribed to the ability of a surface to prevent ice formation or facilitate its detachment by decreasing the adhesion strength between the ice and the surface. In order to develop a material to be considered icephobic, it is key to understand the physicochemical mechanisms involved in the icing of water.

### 2.1. Ice Formation and Accretion in Nature

Icing is a complex process that occurs ubiquitously in nature and different industrial operations. Ice formation is a phase transformation process that, according to the phase diagram of pure water, can take place from vapor to solid (desublimation), from liquid to solid (freezing), or from vapor to liquid and subsequently from liquid to solid (condensation-freezing). Regardless of the phase transformation route, it always begins with nucleation, a stage where clusters of molecules or embryos reach the critical size to become stable nuclei, which further grow forming ice crystals [7,32,33]. Even though the physics and kinetics of ice formation and accretion are out of the scope of this review and further information can be found in the literature [33,34,35,36,37], some basic concepts will be clarified herein. 

There are different types of ice (without considering the crystal structure) that can be found in nature depending on the atmospheric conditions (Table 1). This in turn affects ice properties such as density, hardness, or the adhesion to a given substrate. 

The so-called atmospheric icing is usually classified in three categories: precipitation icing (i), in-cloud icing (ii) and hoar frost icing (iii). 

#### 2.1.1. Precipitation Icing

Precipitation icing, from either snow or rain, is associated with very high accretion rates and consequently a high potential to cause damage. It can be freezing rain, which occurs when rain falls on a surface below 0 °C, resulting in ice with a high density and adhesion. The other type of precipitation icing is snow, which can be either dry or wet snow. Dry snow can become a hazard by accumulation on some specific areas, such as decks or offshore platforms. Its adhesion is initially low, but it can be hard to remove if it partially melts and refreezes again [27]. Wet snow occurs when the air temperature is between 0 and 3 °C and falling snow is slightly liquid (wet snowfall). It shows low adhesion at first, but it can be very difficult to remove from a surface upon freezing. Precipitation icing causes a lot of damage in maritime transport [27], wind turbines [25] and civil structures such as bridge cables [38] or power lines [11]. 

#### 2.1.2. In-Cloud Icing

The second type of atmospheric icing is in-cloud icing, which is characterized by supercooled droplets that hit a cold surface and freeze upon impact. If the airborne water droplets freeze immediately, rime is formed (Table 1), typically at temperatures between −40 °C and −10 °C. If the water droplets that impact the surface do not freeze immediately, a liquid film is formed before freezing and glaze is observed (Table 1). This occurs at temperatures between −18 °C and 0 °C. In-cloud icing is the type of atmospheric icing with more impact in aviation [20], also representing a serious concern for wind turbine blades [9].

#### 2.1.3. Hoar Frost Icing

The third type of atmospheric icing is hoar frost icing, which results from the direct desublimation of water vapor deposited onto cold surfaces. Frost forms in windless or low wind conditions, so it is not a big concern for the aviation or wind turbine blades industry. However, it is present in large exposed areas of ships and offshore platforms, where even a very thin layer of frost (e.g., 0.05 mm) can produce a slipping hazard [27]. 

#### 2.1.4. Sea Spray Icing

In addition to atmospheric icing, sea spray icing is worth mentioning, as it is also responsible for ice-related hazards associated with marine transport industry and offshore platforms, in conjunction with the precipitation icing [39]. Sea spray icing occurs when the seawater drops impact the vessel or offshore platform structure below the freezing point of seawater. The sea spray can be generated either by strong winds that lift the water droplets up to few dozens of meters above the sea surface, or by the interaction of the waves and an offshore platform or a ship. Usually, sea spray icing occurs when the wind speed exceeds 9 m/s, the air temperature is below −2 °C and the seawater temperature below −5 °C, very common conditions in extremely cold regions such as the Bering Sea, the Gulf of Alaska, the Arctic, and most of the Canadian waters [40]. Atmospheric precipitation in the form of freezing rain, supercooled fog and pellets of wet snow or ice is principally responsible for icing in ships and offshore superstructures above 15 or 20 m high, while sea spray icing is the main icing mechanism below that height [39,40]. 

### 2.2. Icephobic Performance and Measurement

As previously stated, a surface is considered icephobic as long as it has the ability to prevent ice formation or facilitate its detachment. Accordingly, there are several specific methods to evaluate and compare the icephobicity of a passive anti-icing surface. It can be based on the capacity to lower the ice adhesion and thus facilitate the ice detachment, delay freezing time, decrease the nucleation temperature or promote the early removal of supercooled water droplets, in order to enhance the chances of supercooled water removal before it freezes and accretes onto a surface [37].

#### 2.2.1. Ice Adhesion Strength 

If the conditions are extreme enough, the ice is going to form and accrete on a given surface regardless of its characteristics. Thus, a very common method to evaluate the icephobicity of a surface consists of measuring the force required to detach the ice from the surface. Here, it should be highlighted that a surface is considered icephobic if the ice adhesion strength (IAS) is below 100 kPa, whereas an IAS below 10 kPa is highly desirable, as this low adhesion allows the natural detachment of the ice by the action of its own weight, the wind or a mild vibration [41]. The ice adhesion measurements rely on the determination of the force required to detach an ice block from a given surface, and can be obtained from Equation (1):

*τ_ice_* = *F*/*A* (1)

where *τ_ice_* is the shear stress required to detach the ice block, usually expressed in kPa, *F* is the maximum applied load in kN and *A* is the apparent contact area between the ice and the solid at the beginning of the test in m^2^. As the main methodology for measuring the ice adhesion strength between an ice block and a given surface is via the application of a shear stress, *τ_ice_* is commonly referred as the IAS of the surface. The interactions between the solid surface and the ice, which are the result of a combination of electrostatic forces, hydrogen bonding, van der Waals forces and mechanical interlocking [31,42,43], can be studied by means of the work of adhesion (*W_a_*) using the Young–Dupré equation:

*W_a_* = *γ_LV_* (1 + *cosθ*) (2)

where *γ_LV_* is the surface tension in air and *θ* is the water contact angle (WCA). Experimentally, it has been observed that the work of adhesion correlates better with the IAS when the receding contact angle (*θ_rec_*) is used instead of the static contact angle [44]:

*W_a_* = *γ_LV_* (1 + *cosθ_rec_*) (3)

Considering Equation (3), a low IAS is expected from materials with low surface energy and high *θ_rec_*. Consequently, superhydrophobic surfaces (SHSs) are the primary icephobic candidates, but it has been demonstrated that they usually fail after a number of icing/deicing cycles, especially under frost formation conditions [45,46]. The fact that SHSs are not always icephobic relies on the fact that the mechanisms of water and ice adhesion are not the same [47]. Ice detachment from a solid occurs through fracture and thus the mechanisms of crack formation, crack propagation and initial length should be taken into account. Thus, it has been observed that IAS is enhanced with the surface roughness for different types of materials [48], as the mechanical interlocking is promoted. Similarly, it is well known that the application of low-surface-energy chemicals tends to decrease the IAS [48]. Actually, very low values of IAS (i.e., lower than 20 kPa) can be achieved as long as there is a low energy mechanism of crack propagation at the ice–solid interface, even if the material is not hydrophobic [49].

**Figure 1:**
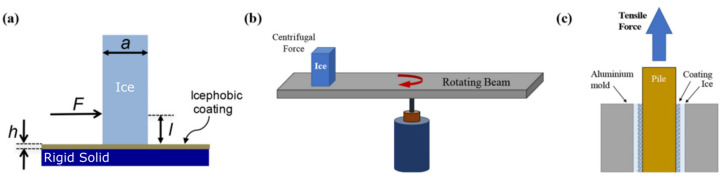
Scheme of the different common configurations to measure the ice adhesion strength in shear force (mode II): (**a**) Peak force method; (**b**) Centrifugal force method; (**c**) Tensile force method. Reprinted with permission from [7]. Copyright (2019) Elsevier.

Among the different configurations to measure the IAS, the most commonly used apply the force to detach the ice in mode II (Figure 1). It is worth mentioning that there are other apparatus that work in mode I (tensile force test in 90° configuration) and even mode III (Raraty and Tabor’s rotational out-of-plane shearing apparatus) [50]. One of the issues to overcome regarding the IAS determination is the lack of a standard procedure, which hinders the direct comparison as the values obtained might differ depending on the ice adhesion measurement technique used, the ice accretion method or the set-up temperature [7,50,51,52]. In fact, it is common to present the results of IAS in terms of an ice adhesion reduction factor (ARF) [53,54,55], where the IAS of a given material is compared with a reference value (i.e., the IAS of the substrate with no coating), so it can also be found in the literature as normalized adhesion strength [56]. The ARF is calculated as the ratio of the IAS reference value to the IAS of the icephobic coating [54], so the higher the ARF, the better the icephobic performance. The most common methods to measure the IAS are schematically shown in Figure 1.

In the peak force method (Figure 1a), ice is grown on a cuvette placed on the icephobic surface to be tested at a given subzero temperature, by pouring water dropwise. The maximum force required to detach the ice is measured and the IAS is obtained by dividing the maximum force applied between the surface area in contact between the ice and the icephobic surface, usually expressed in kPa, according to Equation (1). The load can be applied either by pushing or pulling, but always at a position very close to the surface in order to avoid mixed modes. This method is by far the most used due to the simplicity of the equipment required. The most common set-up is horizontal and applies the load by pushing [44,57], but it can also work vertically [52,58] with the load applied by pulling [49,59]. It is recommended to apply the load at a shear rate of 0.1 mm/s and around 3 mm above the icephobic surface [7], since a higher distance will result in a mixed mode [60]. 

The lack of standard method and commercially available equipment has triggered the custom-built apparatus, where several modifications can be found. For instance, Nguyen et al. [61] included a load cell on a freezing delay apparatus, thus measuring the IAS of the iced droplet instead of using a cuvette to grow an ice block. A similar custom-built test apparatus was used by Zou et al. [62]. Here it should be highlighted that, according to Equation (1), the required force to detach the ice is directly proportional to the surface in contact between the ice and the solid, but the error of a load cell is typically higher at very low loads. Materials with a very low interfacial toughness with ice have shown a linear increase in the force required to detach the ice with the ice–solid contact area up to a critical value (usually few centimeters), where a steady state is observed [63]. On top of that, icephobic surfaces are expected to have a low IAS, so it is always recommended to use an IAS tester set-up where the area in contact between the ice and the solid (A) is at least one square centimeter. 

In the centrifugal force method (Figure 1b), commonly referred as the centrifugal adhesion test (CAT), the icephobic surface to be tested is placed on the tip of a wing or a rotating beam and the ice is grown either with a cuvette [55] or through a freezing rain of supercooled droplets [52]. A counterweight is attached on the opposite side of the rotating wing/beam to balance the system, which is rotated at a gradually increased rotational speed until the ice is detached. The centrifugal force at the time of detachment is estimated as *F = mrω*^2^, where *m* is the mass of ice, *r* is the beam length and *ω* is the speed of rotation at the detachment expressed in rad/s. Once the centrifugal force is obtained, Equation (1) is used to obtain the IAS in the same manner as in the peak force method.

The tensile force method in shear force mode apparatus (conducted in the 0° cone test configuration) consists of two concentric aluminum cylinders with a given gap between them, where the inner surface of the large cylinder is coated with the icephobic material (Figure 1c). The ice is formed between the two cylinders and the ice is detached by using a tensile machine [64]. The maximum pulling force required to detach the ice is obtained and Equation (1) is used to calculate the IAS. One of the drawbacks of this method is that it requires the application of the icephobic coating at the inner part of a cylinder, which might not be convenient for some processing procedures, so it is less commonly used. 

Regardless of the type of IAS tester, the detachment of the ice occurs through fracture, and that fracture can be the response to an adhesive failure at the ice–solid interface or a cohesive failure, either at the ice or at the solid side. On top of that, some mixed situations are also possible if the strength of the ice–solid interface is similar to the cohesive strength of the ice or the solid. If the detachment occurs through cohesive failure at the ice, it is indicative of a very strong interaction between the solid and the ice, tougher than the ice itself, as is commonly the case in ice formation and accretion on bare steel or aluminum, usually showing IAS values above 600 kPa [44,52]. The detachment can also occur through cohesive failure but at the solid, which is indicative of poor mechanical properties of the solid. This can happen at the microscopic level at the outermost surface of the solid and mixed with areas where the failure is adhesive at the ice–solid interface. If the test is repeated the same situation might occur, but a different area of the surface may be damaged by the icing/deicing cycle and ice detachment. Under these circumstances, an increase in the IAS is observed with the number of icing/deicing cycles. Actually, it is common to assume that the resistance of an icephobic coating is demonstrated as long as a similar value of IAS is observed after several icing/deicing cycles [55,57,65,66,67]. Consequently, a stable value of IAS irrespective of the number of icing/deicing cycles is indicative of a pure adhesive failure at the ice–solid interface. This can be considered a first condition of durability; but considering the harsh outdoors conditions that the icephobic coatings might have to face, a stable value of IAS does not guarantee the resistance required for a given application. In the following sections, we will discuss different methods to determine the resistance of an icephobic coating, beyond not being damaged after several icing/deicing cycles and the repetitive adhesive failure at the ice–solid interface, which usually occurs at very low loads, as herein surfaces with icephobic behavior are discussed. 

#### 2.2.2. Freezing Delay Measurements 

**Figure 2:**
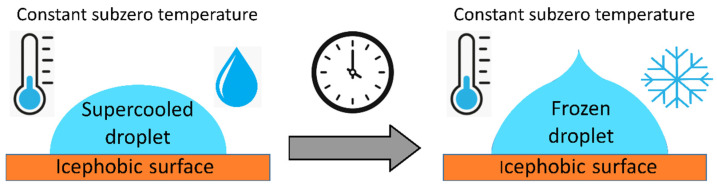
Delay freezing time of supercooled water droplets measured at a constant subzero temperature.

Supercooled/supersaturated water droplets that freeze on a surface are one of the main mechanisms of ice formations and accretion, with special impact on aircrafts and wind turbine surfaces. Thus, the development of a material that can delay the freezing of these droplets is an effective icephobic strategy, with the aim of letting the water droplets run out of the surface before freezing onto it. This delay can be determined by measuring the time elapsed from the first droplet impact on a subcooled substrate to the onset of complete freezing at a given temperature, as can be seen in the scheme of Figure 2. 

**Figure 3:**
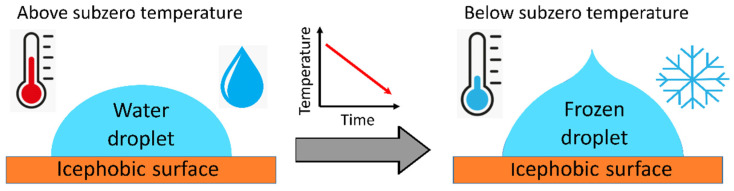
Delay freezing temperature of water droplets measured at a constant cooling rate.

The freezing delay of a water droplet on a surface can be addressed by monitoring the time of freezing onset of complete freezing at a given subzero temperature [68,69] (Figure 2) or by determining the freezing temperature of the droplet under a progressive cooling process [70,71] (Figure 3).

As briefly explained in Section 2.1, nucleation is the first and critical step of the crystallization process, which is explained through the classical nucleation theory [7,32,33]. Nucleation is promoted by the presence of foreign bodies, as the heterogeneous nucleation barrier is always lower than the homogeneous nucleation barrier [33,37,72]. Thus, a supercooled/supersaturated droplet deposited on a surface starts freezing at the interface with the solid, and an ice–water interphase front moves forward until the complete freezing of the drop, forming an upper tip [34], schematically shown in Figure 2 and Figure 3. The formation of this characteristic upper tip shape allows the easy comparison of the time required to observe the complete freezing of a supercooled/supersaturated droplet under some specific conditions rather than comparing any other stage of the freezing process. Similar to the IAS determination, there is a lack of standard and the freezing process of a supercooled/supersaturated droplet is influenced not only by the physicochemical properties of the icephobic surface, but also by the droplet size and initial temperature, the temperature of the icephobic surface and the surrounding atmosphere and the environmental relative humidity [73] affecting the ice crystallization mechanism and consequently the icephobicity of a surface. 

In addition to some controversy originated by the influence of the specific testing conditions, it has been observed that the best freezing retardant behavior can be expected from smooth and hydrophobic surfaces (90° ≤ WCA ≤ 150°) [72], as superhydrophobic surfaces (SHSs, WCA > 150°) may not necessarily offer the best choice for icephobic applications [69,74]. In terms of thermal conductivity, a thermal insulator may further retard the freezing of a supercooled droplet, but its influence is limited [72]. The main two strategies to delay freezing time are based on the use of low-surface-energy chemicals to change the surface energy and wettability, and the use of chemical/physical methods to change the surface roughness and topography [37]. 

#### 2.2.3. Wettability and Related Measurements

The interaction of water with a solid surface differs from the interaction of the same surface with ice. However, as water is the liquid form of ice, it is commonly present during ice formation and accretion mechanisms, as seen in Section 2.1. It is therefore natural to expect icephobic properties from a surface with the capability of repelling water. Actually, a direct relationship has been observed between wettability and IAS [44], as well as with freezing delay [72]. Even though water wettability is not a direct measure of the icephobic performance of an icephobic surface, it is commonly reported for icephobic materials, and in some cases it is provided in order to demonstrate that the surface was not severely damaged after some durability assessment tests, such as a sandpaper abrasion test [75,76,77,78], immersion in solutions at different pH [76,78], cycles of tape-peeling tests [76,77] or UV irradiation time [77]. 

**Figure 4:**
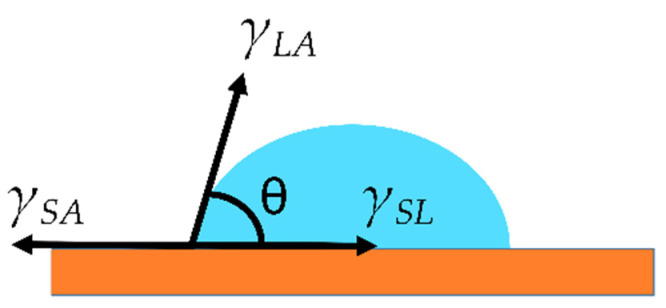
Equilibrium state of a water droplet on a surface where the surface energies are balanced and a static WCA can be measured.

Water wettability can be defined as the tendency of water to spread or adhere on a solid surface. When a water droplet is deposited on a surface, the relative adhesive/cohesive forces determine the shape of the droplet at the different phases/interfaces. The static WCA (*θ*) can be measured with a goniometer when the force balance reaches an equilibrium state (Figure 4), as expressed by Equation (4):

Σ*F_x_* = *γ_SA_* − *γ_SL_* − *γ_LA_* · *cosθ* = 0 (4)

where *γ_SA_* is the surface tension at the solid/air interface, *γ_SL_* is the surface tension at the solid/liquid interface, *γ_LA_* is the surface tension at the liquid/air interface and *θ* is the static WCA, as shown in Figure 4. 

Equation (4) can be reorganized resulting in the Young equation:

*γ_LA_* · *cosθ* = *γ_SA_* − *γ_SL_* (5)

The force balance required to measure the static WCA could be broken either by tilting the goniometer stage or by increasing/decreasing the volume of the water droplet with a microsyringe. This allows measuring the advance WCA (*θ_adv_*), receding WCAs (*θ_rec_*) and WCA hysteresis (CAH, Δ*θ*), as the difference between them. These values are commonly provided, since a positive correlation has been observed between low Δ*θ* and icephobicity [55], whereas it is clear that hydrophobicity is not necessarily linked to icephobicity. Similarly, a direct correlation has been observed between a low IAS and a small water adhesion force [79].

If the static WCA is below 90°, the surface is considered hydrophilic; if it is between 90° and 150°, it is considered hydrophobic; and if it is above 150°, it is considered superhydrophobic. It is well known that lowering the surface energy of a flat smooth surface can give rise to a WCA that cannot exceed 120°; to prepare SHSs, the surface must have a suitable roughness [80,81]. On the other hand, the hydrophobicity of a surface can be enhanced by surface texturing techniques such as surface laser texturing [82].

**Figure 5:**
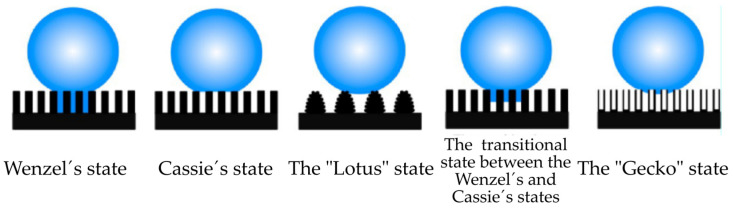
Different superhydrophobic states. Adapted with permission from [81]. Copyright (2016) American Chemical Society.

The appropriate combination of a nano/microstructured surface with low surface energy allows us to achieve SHSs with different water/solid interfaces, as shown in Figure 5. 

Tilting the goniometer stage allows us to measure the roll-off angle, also known as the sliding angle (SA), defined as the angle at which a water drop rolls off, generally below 5° for SHSs [83]. From an icephobic perspective, a water droplet that can slide away from a surface at a very low SA is of interest as the chances that it freezes on the surface before being shed out are minimized. Unfortunately, the sliding angle is generally provided at room temperature as part of a wettability characterization, even for icephobic materials [84,85]. 

**Figure 6:**
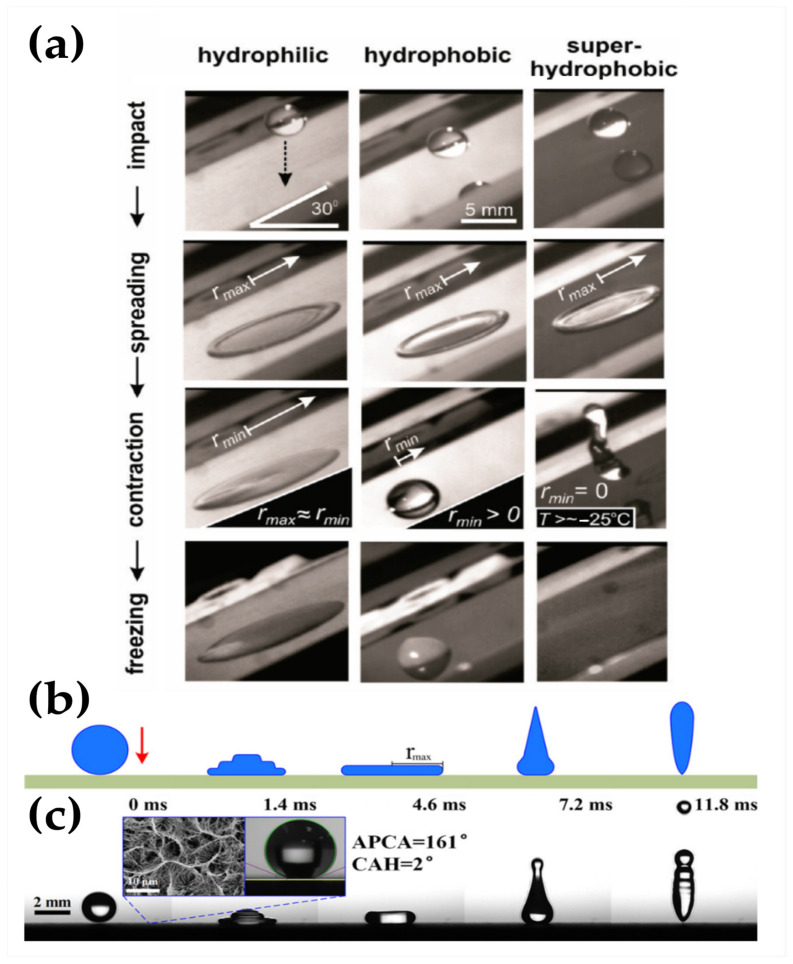
Water droplet impact: (**a**) Droplets impact a hydrophilic, hydrophobic and superhydrophobic surface at temperatures close to −25 °C. The droplets are able to fully retract and shed before freezing on the surface only on the SHS. Reprinted with permission from [87]. Copyright (2010) American Chemical Society; (**b**) Scheme of the bouncing mechanism on a flat superhydrophobic surface; (**c**) Images of the bouncing process captured with a high-speed camera. Reprinted with permission from [88]. Copyright (2015) AIP Publishing.

Following wettability studies under dynamic conditions, the study of the impact of a water droplet is of the utmost interest for icephobic materials, especially at subzero temperatures, given the mechanisms of ice formation and accretion that occur in nature, such as freezing rain or in-cloud icing. When a water drop impacts on a solid surface without wetting it, as it is the case for SHSs, it bounces with remarkable elasticity [86]. As can be seen in Figure 6a, only on SHSs is a water droplet able to impact the surface, retract and bounce off the surface leaving no residue at all even at temperatures as low as −25 °C [87]. When a water droplet impacts a surface, it first spreads to a maximum deformation. If the surface is hydrophobic, the droplet is able to retract. This retraction process can continue until the water droplet bounces off the surface only on SHSs [88], as illustrated in Figure 6b,c. The first spreading stage is mainly driven by the inertia and takes about 20–30% of the total contact time. On the contrary, the retraction process is mainly dominated by the viscous force at the solid/liquid interface [89]. 

The deformations of the droplet during the impact over a superhydrophobic surface are usually studied by means of the Weber number (*W_e_*). This dimensionless number is the ratio of droplet inertia to the surface tension [16,86,89], as expressed in Equation (6):

(6) $$W_{e}=\frac{\rho V^{2}D_{0}}{\gamma_{LA}}$$

where ρ, *V* and *D*_0_ are the density, velocity and diameter of the water droplet, respectively. The greater the value of *W_e_*, the larger the deformations that occur during the impact. An important parameter is the contact time, proportional to the inertia of the droplet, which can be considered as a free oscillation system with stiffness *γ_LA_* and mass $\rho D_{0}^{3}$. The contact time can be approximated by Equation (7) [16,86,89]:

(7) $$t\approx \sqrt{\frac{\rho D_{0}^{3}}{\gamma_{LA}}}$$

**Figure 7:**
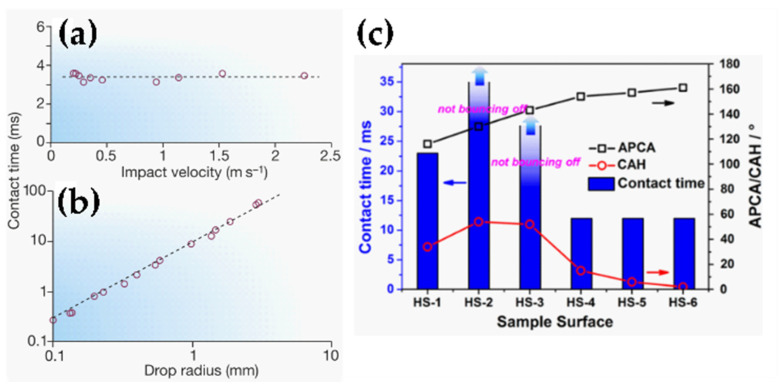
Contact time of a bouncing drop as a function of impact velocity (**a**,**b**) and drop radius (**b**). Reprinted permission from [86]. Copyright (2002) Springer Nature; (**c**) Influence of surface wettability properties on the contact time of a bouncing drop. Reprinted permission from [90]. Copyright (2015) American Chemical Society.

According to Equation (7), the contact time is independent of the initial velocity. This could be counterintuitive, as one could expect a water droplet with a higher velocity to bounce off a superhydrophobic surface more quickly (shorter contact time) than a slower water drop. Even though the drop velocity might drastically affect the extent of deformations, as it affects the Weber number (Equation (6)), it has been demonstrated empirically that the contact time remains constant over a wide range of velocities (20–230 cm/s), as can be seen in Figure 7a [86]. The same authors demonstrated that there is a linear increase in the contact time with the drop radius for all the explored intervals (*W_e_* in the range 0.3–37) [86], as shown in Figure 7b.

Shen et al. [90] studied the relationship of the contact time of an impacting droplet on SHSs with the wettability properties of the surface, such as the apparent WCA and Δ*θ.* They observed that the wetting hysteresis plays a significant role on the contact time (Figure 7c). Bahadur et al. [91] developed a physics-based model for ice formation for a water droplet impinging structured SHS at freezing temperatures that considered contact time, heat transfer and heterogeneous nucleation theory. It was concluded that a water droplet would bounce off a supercooled SHS as long as the retraction force was positive throughout the retraction stage. If the retraction force becomes zero or negative during the retraction stage, ice crystals will nucleate at the tips of the SHS microstructure (solid/liquid interface), causing a further decrease in the retraction force of the impacting droplet that eventually will lead to incomplete retraction, pinning and complete freezing of the droplet. It was observed that the retraction force is strongly affected by the interface temperature, which is in turn mostly affected by the substrate temperature rather than the water droplet’s temperature [91]. In addition to this, lower droplet temperatures enhance the droplet viscosity, which increases the contact time and thus reduces the probability of bouncing [92]. It has been observed that the contact time can be reduced either by using macrotextured SHSs that promote a non-axisymmetric retraction [93], SHSs patterned with lattices of submillimeter-scale nanotextured posts where the drop leaves the surface in a flattened pancake shape without retracting [94] or by using an elastic superhydrophobic film, at which the saucer bounce phenomenon has been observed at an impact velocity of 1.549 m/s, corresponding to a W_e_ slightly above 40 [95]. Some authors have tested the droplet impact and observed drop bouncing at sub-zero temperatures [87,95,96,97,98]. It should be noted that the ability to withstand droplet impact at high velocity (high Weber number) is an indication of mechanical resistance. 

#### 2.2.4. Approximations to Real Icing Scenarios

In order to evaluate the anti-icing performance of an icephobic surface, some authors have simulated several icing scenarios. Ice accumulation tests recreate ice formation and accretion conditions in a controlled manner, and subject both the icephobic surface to be tested and an untreated reference material. Icing wind tunnels have been used to recreate precipitation icing or in-cloud icing conditions [80,99,100,101], while other authors have used different set-ups to recreate sea spray icing conditions [54,102], freezing rain [103,104], soft dripping of cold water (0 °C) over the icephobic surface at subzero temperatures [77] or environmental cabinets in which both temperature and humidity can be accurately controlled, allowing us to measure ice accretion rates at different angles [71,105,106].

It is also common to subject the icephobic surfaces under study to outdoor icing conditions [57,107,108,109]. It can be performed for a relatively long period, becoming simultaneously a durability test. For instance, Golovin et al. [57] tested simultaneously the icephobicity and durability of different icephobic coatings by placing them outdoors for 4 months during winter in Michigan, USA. Other researchers tested the icephobic coatings for very long periods (almost 30 months, including three Russian winter seasons, with temperatures as low as −17 °C) [108] or just made a comparative outdoor test for a single icing event (e.g., heavy snowfall at −3 °C, relative humidity 99%, and wind velocity of 2 m/s) [109]. 

## 3. Durability of the Icephobic Coatings: Resistance Assessment

Icephobic materials are intended to be used under harsh conditions beyond extremely cold temperatures, including mechanical abrasion, sand or droplet impacts at high velocity, contact with corrosive chemicals or long-term UV exposure. In order to ensure the suitability of an icephobic surface for a specific application, it is important to study its durability. Given the variety of harsh environments an icephobic surface might be intended to withstand, there are many different tests to determine its resistance against harsh conditions, which can be typically classified in three broad groups (mechanical, chemical and environmental methods), even though sometimes the same test could be included in more than one category.

**Figure 8:**
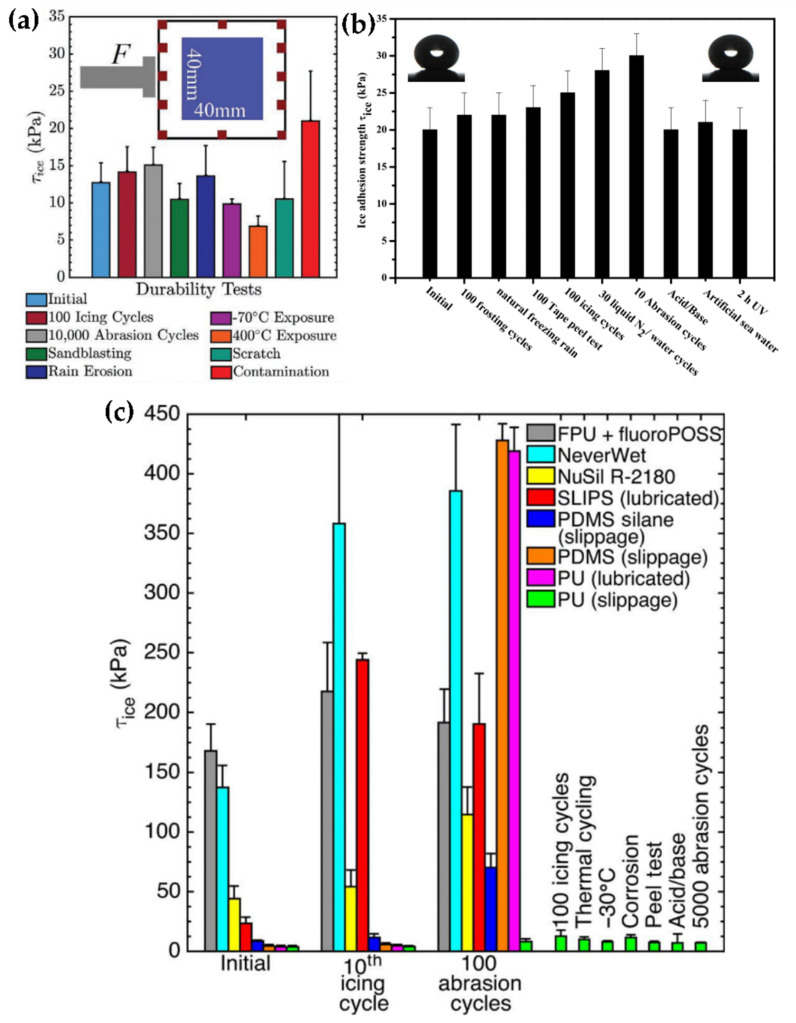
Ice adhesion strength after several durability tests: (**a**) Icephobic performance of buckling elastomer-like anti-icing metallic surfaces after durability tests. Reprinted with permission from [110]. Copyright (2022) John Wiley and Sons; (**b**) Icephobic performance of candle soot coating after different mechanical tests. Reprinted with permission from [107]. Copyright (2019) American Chemical Society; (**c**) Icephobic performance of different icephobic surfaces after several durability tests. Reprinted with permission from [57]. Creative Commons CC BY-NC (2016) by Golovin et al.

In spite of the importance of the durability of an icephobic coating, the resistance of an icephobic surface is studied in depth very seldom, where some remarkable works are worth mentioning as exceptions [57,107,110]. In Figure 8, the IAS of different icephobic surfaces is shown after several durability tests, where the myriad of different procedures to test the resistance of an icephobic coating can be observed at a glance. 

As will be discussed in the next section, there are different approaches to provide icephobicity to a surface, and depending on the type of icephobic surface or targeted application, one durability test or some specific conditions might be more suitable. In addition, due to the lack of standards for many specific tests, the testing conditions vary a lot from one laboratory to another, making broad comparisons difficult. 

### 3.1. Mechanical Methods to Assess the Durability of an Icephobic Coating

#### 3.1.1. Icing/Deicing Cycles 

It is very common to address the durability of an icephobic surface by determining its icephobicity several times. Usually, the IAS is determined over the same icephobic area at least 10 or 15 times and plotted over the number of icing/deicing cycles [59,67,111,112,113,114,115,116,117,118,119]. If the icephobic surface is damaged during the detachment of the ice block and there is no self-healing mechanism, this damage will be revealed by an increase in the IAS after very few icing/deicing cycles. On the one hand, this method is very simple, not time consuming and does not require any specific sample preparation. On the other hand, its main drawback relies on the fact that only very weak icephobic surfaces fail after a few icing/deicing cycles. 

#### 3.1.2. Sandpaper Abrasion Test

**Figure 9:**
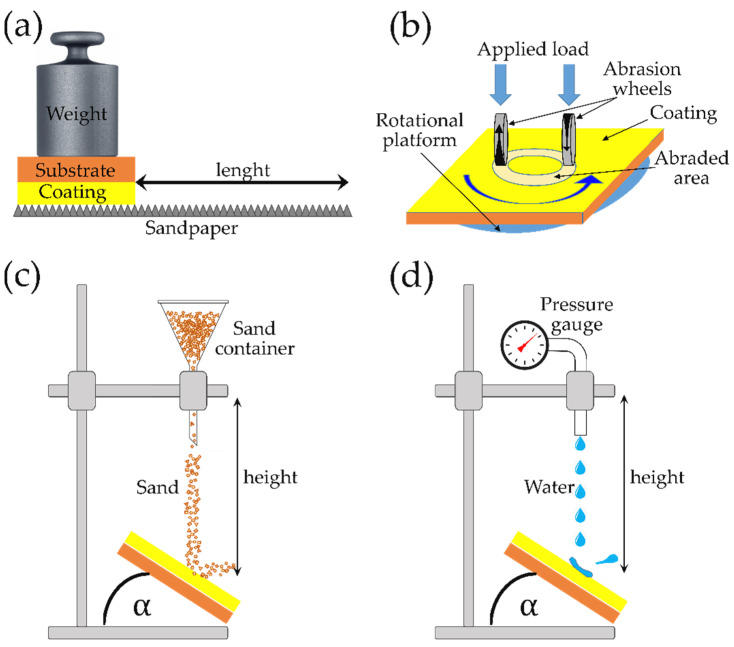
Schematic illustration of common mechanical methods to assess the durability of an icephobic coatings: (**a**) Sandpaper abrasion test; (**b**) Taber abrasion test according to ASTM D4060 [124]; (**c**) Sand impact test; (**d**) Water impact test.

The sandpaper abrasion test consists of damaging the icephobic surface with sandpaper, applying a constant and controlled pressure with a weight, for a given length, usually in one direction to and fro, as can be seen in Figure 9a. This type of test can be automated using a linear abrader [120]. The lack of standard hinders the direct comparison between icephobic materials and promotes the use of self-designed tests. The sandpaper can be as coarse as a 80 grit sandpaper [49] or as soft as a 10,000 mesh sandpaper [121]. In addition to this, there is also a wide variety in the applied pressure and the test length [49,65,66,67,107,121,122,123]. To visualize the effect of the sandpaper abrasion on the icephobicity of a surface, the IAS is generally plotted versus the number of abrasion cycles, although for comparison it is more adequate to use the total distance. Instead, some authors have reported the worn-down thickness [66]. 

#### 3.1.3. Taber Abrasion Test

The Taber abrasion test was developed to determine the resistance of organic coatings to abrasion, as described in the ASTM D4060 standard [124]. Briefly, the coating to be tested is mounted on a rotational platform and two abrasive wheels are pressed against the coating, as can be seen in Figure 9b. The rotational speed is kept constant at 60 or 72 rpm and the force of the wheels can be precisely adjusted by a set of weights, from 125 g [125] to more than 1 kg [57]. This standardized method was used for different types of coatings [126,127] before it was used to test superhydrophobic [125,128] or icephobic surfaces [49,57,110,129]. The wear index can be calculated as the ratio of weight change to the number of cycles, with it also being common to report the weight loss or thickness reduction as a wear indicator. Even though ideally the IAS should be measured after several Taber abrasion cycles [129], or at least after a high number of Taber abrasion cycles [57,110], the size of the groove (width typically below 15 mm) makes it difficult to determine the IAS with some IAS testers and sometimes only wettability data are provided [49]. 

#### 3.1.4. Sand/Water Impact Test

The erosion tests based on the study of the effect of sand impact on the icephobic surface are schematically shown in Figure 9c. Typically, sand of a specific grain size falls from a given distance and impacts the icephobic surface at a certain angle α. The lack of standards gives rise to a variety of testing conditions. The severity of the test can be evaluated by the comparison of the impulse force of the sand particles (*J*) that impinge on the surface. Assuming that *J* should be equivalent to the change in momentum [130], Equation (8) can be used:

*J* = *mv*/Δ*t* (8)

where *m* and *v* are the mass and velocity of the impacting sand particle, respectively, and Δ*t* is the dwell time on the surface. Some authors have used relatively soft conditions (sand of 100–300 mesh falling from 50 cm) [131], whereas others have chosen to conduct a more aggressive test (sand of 0.5–1.0 mm falling from 90 cm) [132]. Even though providing the IAS (or any other direct icephobic performance measurement after the erosion test) is the most suitable measurement from an icephobic perspective [106,131], it is also common to report other data that allow us to evaluate the damage of the surface such as WCA and SA [132], or the weight loss after the test [106,111,132]. This type of test is also referred to in the literature as the sand erosion test [16,106], sand dropping test [131] or falling abrasive test [132].

The water impact test is commonly very similar to the sand erosion tests, where the impingement material is the only remarkable change, as can be seen in the scheme shown in Figure 9d. In the same way, the severity of the test can be modulated by tuning the testing conditions. Sarma et al. [117] conducted the water impingement test by holding the icephobic samples under a water faucet (flow rate of 0.2 L/s) for 30 min while Irajizad et al. [53] exposed the samples to a high shear flow of water (and air) for one month. If the water drop speed is very high, the test is commonly referred as rain erosion, e.g., in the works of Rico et al. [111] impinging water droplets of 2 mm at a raindrop speed up to 225 m/s or Alasvand Zarasvand et al. [110] using a water jet at a flow rate of 5.49 l/min at a pressure of 9.3 MPa. Other authors have used HCl instead of pure water to simulate acid rain [131,133]. 

There are other water/sand impact test modifications that are worth mentioning, including the use of sand blasters [106,134] to enhance the velocity of the sand particles or the addition to the water jet of an abrasive agent such as SiC microparticles to impinge silicon carbide suspensions at high speed [135,136]. 

#### 3.1.5. Tape-Peeling Test

This method is based on the placement of a conventional adhesive tape on the surface to be tested, the subsequent application of certain pressure, and finally peeling the tape off. This is repeated as many times as desired and after some cycles the effect on the icephobicity is evaluated, ideally by directly measuring the IAS [107], or indirectly by wettability measurements, such as WCA and SA [77,109,114]. The latter is very common for icephobic surfaces based on the superhydrophobicity behavior, as they usually suffer from poor mechanical stability, and even a slight scratch or abrasive force is capable of destroying the nano/microstructure that leads to superhydrophobicity.

#### 3.1.6. Wiping Test

A wiping test is usually conducted to test icephobic surfaces that are based on the presence of a lubricating layer at the ice–solid interface to achieve a low IAS. This is typically the case of slippery liquid-infused porous surfaces (SLIPS), where the surface can be wiped with a cloth in order to remove the lubricant and observe the regenerative ability of the coating [137,138]. In some cases, a relatively high number of cycles have been applied, using a cloth [137], a pencil rubber [104] or even a car windshield wiper [117]. 

#### 3.1.7. Mechanical Properties of the Icephobic Coatings

In the vast majority of the durability tests that have been discussed already, the aim of the test is to first subject the icephobic surface to certain conditions with the potential to damage the surface and after that, conduct the subsequent evaluation of the effect on the icephobic performance of the coating or the damage by any other means, such as wettability measurements. In addition to this, sometimes the durability of an icephobic coating is addressed by measuring the mechanical properties of the coating itself. This can be achieved by nanoindentation (ISO 14577-1:2016 [139]), where the elastic modulus and hardness of the coating is provided [106,140,141]. Other authors have used the pencil scratch test (ASTM D3363-22 [142] or ISO 15184:2020 [143]), where the hardness of the coating is addressed by evaluating the damage after dragging pencils of different hardness over the icephobic substrate [66,106,144]. 

#### 3.1.8. Icephobic Coating-Substrate Adhesion 

In addition to the mechanical properties that can be addressed by the test described in the previous subsection, there are other tests devoted to evaluating the coating–substrate adhesion, as it is as important as the mechanical resistance of the coating itself. Some researchers have used the cross-cut adhesion test (ASTM D3359-23 [145] or ISO 2409:2013 [146]) to qualitatively measure the adhesion between the coating and the substrate [66,106,133,141,147,148,149], while others have used the dolly push-off test (ASTM D4541-22 [150] or ISO 4624:2016 [151]) to quantitatively measure the normal force necessary to remove the coating from the substrate [141], or to affirm that it is beyond a certain value when the failure occurs through the coating itself rather than through adhesive failure at the coating–substrate interface [106]. These tests are briefly described in Table 2 among other common tests to evaluate the mechanical durability of an icephobic coating. Other authors have used less common methods to measure the adhesion between an icephobic coating and the substrate such as a bending test [147], ultrasonic agitation [147] or nanoscratch [140]. 

### 3.2. Chemical Methods to Assess the Durability of an Icephobic Coating

The resistance of icephobic coatings against harsh chemical conditions has been commonly tested by soaking the coatings in acid, basic or corrosive media.

#### 3.2.1. Acid, Basic or Organic Solvent Immersion

Many authors have tested the resistance to the icephobic coatings to harsh chemicals by soaking them in acid/basic media and measuring the IAS [53,57,107,131,133,153] or the WCA [134] after immersion, as it is very common to determine the wettability instead of the IAS for icephobic surfaces based on superhydrophobicity. Others soaked the icephobic samples in different pH chemical solutions and just evaluated the integrity of the coatings after immersion [53]. In order to evaluate the chemical resistance of the coatings, organic solvents have also been used. This is the case of Sarma et al. [117], who immersed the coated substrates in toluene, ethanol and acetone subsequently for 2 min each and measured the IAS after dying with compressed air. 

#### 3.2.2. Corrosion Methods

Golovin et al. [57] conducted an accelerated corrosion test according to the ASTM B117-19 standard [154]. Briefly, the icephobic coating is scratched and kept in a salt spray fog chamber at a warm temperature for a long period (i.e., 35 °C for 200 h) and after that the ice adhesion is measured. Other authors soaked the icephobic coating in NaCl (5 M) for 2 h [153] or in artificial seawater [107] and measured the ice adhesion after immersion. In contrast, Momen and Farzaneh [148] measured the corrosion resistance of an icephobic coating and its ability to protect an aluminum substrate, but did not determine the icephobic performance after corrosion. Other authors investigated the corrosion resistance of an SHS through the electrochemical corrosion test (3.5 wt.% NaCl aqueous solution) [77,155] and observed that the icephobic coating was able to protect the steel substrate against corrosion. The protective effect was ascribed to the formation of air pockets on the hierarchical superhydrophobic structure. 

### 3.3. Environmental Methods to Assess the Durability of an Icephobic Coating

Icephobic coatings are expected to withstand extreme environmental conditions beyond subzero temperatures, which includes long-term exposure to UV light, high humidity or thermal cycles. Therefore, some tests have been conducted to mimic specific environmental scenarios and evaluate their effect on the icephobic performance, among which UV irradiation and/or thermal exposure are the most common. 

#### 3.3.1. UV Irradiation Exposure

Some authors have exposed the icephobic coating to UV light and, after some time, measured the IAS value. Irajizad et al. [53] exposed the as-prepared icephobic coating to UV irradiation for 500 h (3 weeks) in a UV chamber with 100% humidity and measured the IAS after the UV exposure. Similarly, Wang et al. [77] exposed the icephobic samples to UV light for 50 h at 50 °C but determined the WCA instead. Other authors subjected the icephobic samples to cycles of UV radiation and moisture exposure. This type of test was followed by Wu et al. [106], who measured the IAS of the icephobic coatings after 144 h (6 days) of cycles of 4 h of UV radiation at 60 °C, followed by 4 h moisture exposure by condensation at 50 °C, following the ISO 11507:2007 standard [156].

#### 3.3.2. Thermal Exposure

Due to the lack of a standard, the testing conditions vary from one laboratory to another, as with other durability testing methods. Alasvand Zarasvand et al. [110] placed some samples in a freezer at −70 °C for 24 h and measured the IAS after it. They also measured the IAS of other icephobic samples after being placed on a hot-plate at 400 °C for 2 h. Previously, Golovin et al. [57] had already followed a similar procedure, measuring the IAS at −10 °C of samples left on a hot-plate at 70 °C for 24 h, repeating the cycle 10 times. Likewise, Sharma et al. [117] set the temperature of the hot-plate at 60 °C and left the samples for 48 h before measuring the IAS for 100 icing/deicing cycles. 

## 4. Icephobic Surfaces and Different Strategies to Enhance Their Durability

A surface is considered icephobic due to its ability to repel ice or mitigate ice accretion, and there are several icephobic strategies that can be followed, an aspect that is commonly used to classify icephobic materials (see Table 3). Nevertheless, the different icephobic strategies and surface design is a topic that has already been addressed in several exceptional reviews [7,30,32,37,60,157,158,159,160,161,162], so herein we will focus on the most representative efforts that have been made to enhance the durability of different types icephobic materials. 

As can be inferred from Table 3, the boundaries between the different types of icephobic materials are often faded and sometimes a specific icephobic material can be classified in more than one specific type. Consequently, the next classification is only intended to provide an overview of the most relevant types of icephobic materials and the different developed strategies to enhance their durability. In addition to the type of icephobic material, where the chemical nature and/or microstructure of the material is considered, it is paramount to observe the different icephobic mechanisms, as recently reported Dhyani et al. [60]. Moreover, each mechanism relies on a different set of mechanics and a corresponding governing equation that may be used to evaluate the ice adhesion force (*F_ice_*), where it represents the required force to remove ice from a surface per unit width (Table 4). In addition to the governing equation, the engineering parameters that can be modified towards the design of an icephobic surface and the test parameters required to measure the ice adhesion strength are also shown. 

### 4.1. Superhydrophobic Icephobic Coatings

If the selected strategy is aimed at reducing the IAS, the main two options are focused on reducing either the work of adhesion between the ice and the coating (*W_a_*) or the shear modulus of the coating (*G*), as the IAS required to shed off a block of ice from a thin film can be predicted by adhesion mechanics, as shown in Equation (9) [57,112,117]:

(9) $$\tau_{ice}=A\sqrt{\frac{G\;W_{a}}{t}}$$

where *A* is an experimental constant and *t* is the thickness of the coating. This equation predicts the shear stress required to cleave two surfaces apart, a phenomenon that occurs through interfacial cavitation [57,60,112,117], as can be inferred from Table 4. If the path of minimizing the work of adhesion is chosen, SHSs seem like appropriate candidates for an icephobic surface, as they minimize the contact between the surface and the ice to be formed through interfacial air pockets, promoting the interfacial cavitation at low shear stress. Thus, when the first synthetic SHS [164] was achieved in 1996 it was a breakthrough point in the development of icephobic materials. However, limitations were soon observed, as SHSs usually lack resistance and cannot retain their icephobic ability under harsh conditions such as high humidity and temperature below the nucleation point [47,74,162]. In addition, the icephobicity of SHSs tends to decrease after a few icing/deicing cycles as the asperities of the surface are very likely to be damaged during icing (water solidification) and/or deicing (water removal) [165]. The IAS of the damaged SHS increases abruptly as the ice detachment occurs through both adhesive and cohesive failure [45]. This occurs when the micro/nanocavities of the damaged textured surface are filled with water, transitioning from a non-wetted Cassie–Baxter state to a wetted Wenzel state [166]. Even though it has been observed that some SHSs can maintain their low IAS for several icing/deicing cycles (Cassie ice), typically additional cycles begin to degrade the surface, rising the IAS [57]. In fact, the mechanical stability of SHSs is the main factor limiting their practical application, including their use as icephobic coatings. Thus, the development of mechanically durable SHSs has drawn a lot of interest in the last decades [167], as the preparation of a highly resistant SHS is a technological and scientific challenge. 

#### 4.1.1. Durable Superhydrophobic Coatings

A superhydrophobic surface can be prepared by applying a coating over the desired substrate, and to ensure its durability the coating should be mechanically stable and strongly attached to the substrate. SHSs have been obtained by plasma spraying [168], the sol-gel method by atmospheric spraying [169,170] and spin-coating [107,171], among others [134,172,173]. Sharifi et al. [168] plasma sprayed a TiO_2_ suspension over grit-blasted stainless steel substrates. The micro textured surface was then treated with stearic acid and an SHS with a WCA of 170° was generated. This coating decreased the ice accretion rate (compared with commercial superhydrophobic coatings) and maintained a high WCA with up to 40 icing/deicing cycles in a wind tunnel. Also, they demonstrated better resistance to dry particle erosion compared with the two commercial coatings. Li et al. [169] sprayed a polyurethane thin layer and subsequently a suspension containing SiO_2_ nanoparticles, tetraethoxysilane (TEOS) and a long chain alkoxysilane, achieving an SHS with a WCA of 163.9° when sprayed over a glass slide. Coated glass slides showed a freeze delay under static conditions (60 μL water droplet, −15 °C, relative humidity of 54%) of ca. 30 min and under dynamic testing no ice was observed. These authors demonstrated the self-healing capability of the coating, as it recovered a WCA above 150° after an O_2_ plasma treatment, which was ascribed to the capability of the long chain alkoxysilane to migrate to the surface. The chemical stability of the coatings was tested in organic solvents, acid, basic and salty aqueous solutions, and the WCA and SA did not show any remarkable change. In addition, the mechanical stability was evaluated by sandpaper abrasion and tape-peeling cycling tests, and the WCA decreased and the SA increased with both tests. Unfortunately, the icephobicity was not evaluated after those durability tests by any means. Jamil et al. [107] spin-coated a commercial silicone adhesive over a glass slide and, after soot deposition (holding the coated glass slide above the flame of a paraffin candle), SHSs were obtained. The carbon nanoparticles showed a weak interaction with the substrate, but the surface adhesion and mechanical stability was enhanced with the use of a binder and a WCA of 158° was observed. The surface repelled supercooled water (−10 °C), maintaining a Cassie–Baxter state and a moderate freezing delay. Moreover, an IAS of about 25 kPa was observed after different severe mechanical tests (Figure 8b). Ruan et al. [171] spin-coated an alumina precursor solution onto aluminum substrates, which were further modified with lauric acid, obtaining an SHS with a WCA as high as 157.6°. The IAS was reduced by half and the icing time was delayed while the freezing temperature was reduced (ice was observed after 15 min at −4.1 °C for the bare aluminum, while it was observed after 65 min at −8.3 °C for the SHSs). Unfortunately, the durability of the coating was not addressed by any means. Hong et al. [155] dispersed fluorinated crosslinked microspheres of poly[hexafluorobisphenol A-co-cyclotriphosphazene] in PDMS to prepare a superhydrophobic coating with WCA of 164° and SA of 3.7°, successfully applied over aluminum, steel, paper or cotton. An impressive freezing delay (40 μL water droplet, −15 °C, relative humidity of 70%) was observed, as the droplets were completely frozen at 28 s on a pure aluminum slide, while it took 1472 s to freeze on the SHS. In the dynamic regime, a water stream was poured from 1 cm height over the samples (−15 °C, relative humidity of 70%) and after a few minutes a large block of ice was formed on the bare aluminum while no ice was observed on the coated surface. In addition, the IAS of the SHS was 60 kPa while the IAS of the bare aluminum was 320 kPa, which accounts for an ARF of 5.3. In addition, the SHS maintains its superhydrophobicity after finger-scratch, sandpaper abrasion (200 grit sandpaper, load of 50 g, 120 cm) and knife-scratch tests.

#### 4.1.2. Two-Step Preparation of SHSs: Roughen the Substrate and Lower the Surface Energy

It is well known that a smooth low surface energy material typically shows a WCA below 120°; therefore, suitable roughness is required to observe superhydrophobicity [80,81]. This takes us to the strategy to achieve mechanically-resistant SHSs [111,173,174] in which the substrate is first nano/microroughened and afterwards the surface energy is lowered. An appropriate nano/microstructure is prepared, usually over a metallic substrate, by laser roughening/lithography [46,111,114,115,175,176], chemical etching [77,109,133,177,178,179], anodizing [133,166,180,181,182], electrochemical [183], plasma treatment [184] or even using a template to replicate the nano/microstructure on the substrate of interest [180]. This second chemical treatment is mostly conducted with low-surface-energy chemicals such as fluorocarbons [109,111,115,133,178,180] and alkoxysilanes [178,179,184] in addition to other less common low-surface-energy chemicals [177,181,183]. The fact that the final micro/nanotextures are created on the substrate itself eliminates interfacial adhesion issues, paramount with the first strategy outlined above. Boinovich et al. [109] deposited hydrophobically-modified silicon nanoparticles on chemically-etched steel surfaces. The SHSs maintained a WCA exceeding 155° and a rolling angle of 42° after 100 icing/deicing cycles. In addition, it was observed that the superhydrophobicity was maintained after both a tape-peeling test (130 kPa) and a mechanical test to evaluate the resistance to cavitation erosion by immersion in an ultrasonic bath (35 kHz, 55 W, 10 min). They also performed an outdoor test (heavy snowfall at −3 °C, relative humidity of 99%, wind speed of 2 m/s) and the treated surface remained almost clean whilst ice and snow accreted onto the bare stainless steel. Wang et al. [77] prepared mechanically stable SHSs by chemically etching a steel substrate and further chemically grafting with 1H,1H,2H,2H-perfuorodecyltriethoxysilane (FAS-17). A water-dripping test was carried out (substrate at −20 °C and water droplets at 0 °C) and no frozen spot could be observed on the SHS whilst the bare steel was covered with ice. A tape-peeling test was conducted and the WCA and SA changed from 160° to 155° and from 1° to 5°, respectively, after 70 peeling cycles (applying a pressure of 31.2 kPa, much larger than the “finger press” required to follow the standard ASTM D 3359-09 [152]). A sandpaper abrasion test was conducted (400 grit sandpaper, 16 kPa, 110 cm) and the roughness (Ra) decreased from 1.323 μm to 0.256 μm, while the SHS maintained its superhydrophobicity (WCA of 152° after sandpaper abrasion). Similarly, the superhydrophobicity was retained (WCA > 150°) after 180 min of water impact test (10 μL water droplets falling from 30 cm at 2.5 m/s) or 50 h of a UV-durability test (UV light of 350 nm, 500 W, 50 °C). Moreover, an anticorrosion property was demonstrated through an electrochemical corrosion test (3.5 wt.% NaCl solution), which was attributed to the presence of air pockets and not to an oxide layer. 

**Figure 10:**
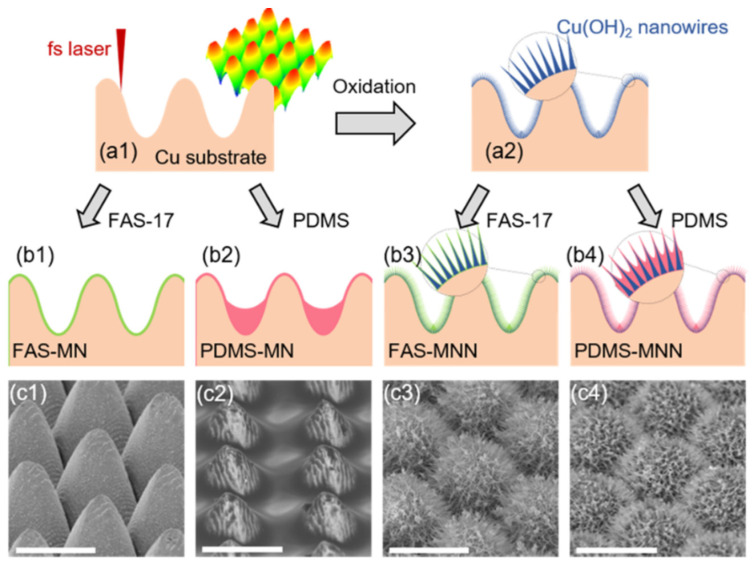
Illustration of the SHSs prepared by Chen et al. [114]. Primary microcone arrays were fabricated by laser ablation (**a1**) and close-packed Cu(OH)_2_ nanowires were formed by subsequent wet chemical oxidation (**a2**); FAS-17 and PDMS are represented by green and red, respectively; (**b1**–**b4**) Scheme and (**c1**–**c4**) SEM images of the four samples. Scale bar is 30 μm; Reprinted with permission from [114]. Copyright (2022) American Chemical Society.

Pan et al. [180] generated a nano/microstructure by sandblasting aluminum sheets followed by anodizing in a phosphoric solution. The rough surface structure was then replicated on a carbon fiber/thermoplastic composite by hot-press and finally the surface of the composite was further modified with heptafluorosilane, obtaining an SHS with a WCA and SA of 158° and 8°, respectively. The SHS showed icephobicity with a freezing delay of 460 s and an IAS of 36.6 kPa. In addition, they evaluated the mechanical stability of the coating with an abrasion test (400 grit sandpaper, 0.8 kPa, 240 cm) and observed that the WCA was still above 150°. Liao et al. [179] chemically etched an aluminum foil substrate by immersion in a CuCl_2_ solution and after the etched foil was modified with hexadecyltrimethoxysilane, obtaining an SHS with a WCA and SA of 161.9° and 6.8°, respectively. The SHS delayed ice nucleation and/or formation while maintaining their superhydrophobicity after water-drop impact (50 μL water droplets, height of 40 cm, impinging velocity of 2.83 m/s) and a sand impact test (210–350 μm in diameter, height of 40 cm). WCA was acquired after 5000 water droplets and 20 g of sand grains. Zheng et al. [182] prepared SHSs by anodizing aluminum plates with H_2_SO_4_ and subsequent treatment with FAS-17, with a WCA and SA of 156° and 2.5°, respectively. The SHS showed a moderate freezing delay and a low IAS (40 kPa). Furthermore, the SHS showed a good mechanical stability against sand erosion. They sandblasted SiO_2_ particles (63 μm in diameter, stand-off distance of 15 cm, pressure of 30 kPa) for 30 s, 60 s and 90 s and a plateau was observed in the weight loss at 30–90 s and, even though the WCA decreased, it remained above 150°. In spite of finding a good stability against sand erosion, the WCA slightly decreased after very few icing/deicing cycles, which was ascribed to partial damage of the asperities of the surface during shearing of the ice. On the other hand, the SHSs demonstrated improved weathering (UV exposure and condensation for 7 days) and corrosion (3.5 wt.% NaCl solution) resistance. Following this strategy, other authors have achieved durable SHSs by creating nano/microstructures with highly resistant nanostructures such as Cu(OH)_2_ nanowires [114] or ZnO nanohairs [185]. Chen et al. [114] prepared an SHS combining laser ablation of copper substrates followed by chemical oxidation to create Cu(OH)_2_ nanowires on the surface that were further modified with FAS-17 or PDMS (Figure 10). The SHSs remained superhydrophobic and icephobic after abrasion tests (1000-grit sandpaper, 1.2 kPa, 300 cm), 60 icing/deicing cycles or 500 tape-peeling cycles. 

**Figure 11:**
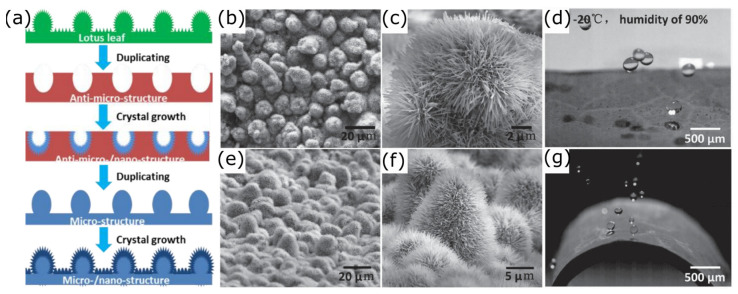
SHS fabricated by Wang et al. [185]. (**a**) Schematic illustration of the flexible PDMS microstructure decorated with ZnO nanohairs; (**b**) Top view and (**e**) side view (tilted angle of 60°) of the flexible SHS; (**c**,**f**) Magnified images of a single papilla covered by ZnO nanohairs from the top and side; (**d**,**g**) Motion of droplets on the flexible SHS over flat and curved samples, respectively. Reprinted with permission from [185]. Copyright (2016) John Wiley and Sons.

Similarly, Wang et al. [185] prepared a nano/micro hierarchical surface by duplicating the negative structure of the lotus leaf with PDMS by soft-lithography and then growing ZnO nanohairs on it (Figure 11). Finally, the hierarchical nano/microstructure was treated with FAS-17 to obtain a low surface energy, similar to that of the lotus leaf. The flexible SHS showed water and ice repellency (−20 °C, relative humidity of 90%) for up to three months and an effective freezing delay time even after 100 bending tests. In addition, water droplets (10 µm, 0° C, 10 m/s) impacted and bounced off the surface without causing any damage. 

The work of Cheng et al. [186] is worth mentioning as they prepared a flexible SHS with soft PDMS microspheres and stiff SiO_2_ nanoparticles sprayed over a semidry fluorinated resin. The pristine surface showed a WCA of 171.3° and an IAS of 1.53 kPa that remained below 7 kPa even after 25 icing/deicing cycles or 100 abrasion cycles (400 mesh sandpaper, applied weight of 200 g and 10 cm to and fro per cycle). The durability of the flexible SHS was attributed to the fact that the surface still maintained a soft and stiff alternating structure with a WCA of 168°.

#### 4.1.3. Self-Healing SHSs

In addition to the strategies already discussed to prepare a resistant SHS, the durability of the coating can be enhanced by preparing a self-healing SHS. Even though this is a very appealing strategy, self-healing studies on SHSs usually present very limited mechanical durability characterization, a lack of anti-icing performance and are mostly focused on WCA data to discuss the recovery of the superhydrophobicity [128]. Fu et al. [132] used a two-step thiol click reaction to prepare fluorinated PU with SiO_2_ nanoparticles (SiO_2_-FPU). The superhydrophobic SiO_2_-FPU coating showed a remarkable icing-delaying performance since a drop-like ice formed after 300 s while it was observed on both fluorinated PU-coated and bare glass slides at around 60 s (30 μL water droplet, relative humidity of 60%, surfaces tightly attached to a −15 °C cooling stage and ambient temperature of 15 °C). The SiO_2_-FPU showed a WCA of 163° and its superhydrophobicity was maintained after a 30 m sandpaper abrasion test (500 grit sandpaper, 10 kPa), 450 tape peeling cycles (pressure of 1 kg) and 1.5 h of water dripping (around 14,400 water droplets of 150 μL, height of 40 cm, impact velocity of 3.1 m/s), but the anti-icing performance was not specifically evaluated after any durability test. The SiO_2_-FPU coating showed self-healing properties as it could recover its superhydrophobicity after chemical etching (in acid or base for 8 h or 2 h, respectively). To recover the superhydrophobicity, the SiO_2_-FPU coating was annealed at 135 °C for 3 h to allow the migration of flexible and branched fluoroalkyl chains, but it was observed that they were consumed in the first two cycles, as the WCA after the third chemical etching/annealing cycle was only 137°. There are many other works on SHSs with self-healing capability [187,188], but usually no icephobic performance is evaluated. 

### 4.2. Icephobic Coatings with Low-Surface-Energy Chemicals

Low-surface-energy chemicals are appropriate to lower the work of adhesion and thus design icephobic coatings according to Equation (9), and, as it has been observed many times, a hydrophobic surface might perform better as an icephobic material than an SHS. Bharathidasan et al. [64] studied the effect of wettability (WCA and SA) and surface roughness on the IAS of hydrophilic, hydrophobic and superhydrophobic surfaces. They prepared a set of coatings on Al substrates using polyurethane (PU) and polymethylmethacrylate (PMMA) for the hydrophilic coatings, two types of silicones (R2180 and RTV11) for the hydrophobic coatings and three different types of superhydrophobic polymer-silica nanocomposite coatings (R2180-EH5, RTV11-EH5 and PMMA-HMS). They observed a low IAS on smooth hydrophobic silicone coatings (ARF between 25 and 43) as compared to the rough superhydrophobic coatings (ARF between 1.3 and 4.4), which performed slightly better than the hydrophilic coatings (ARF between 1.0 and 1.3). It was concluded that smooth surfaces with a low surface energy are a good choice for icephobic materials with a low IAS. 

#### 4.2.1. Fluorinated

If the use of low-surface-energy chemicals is considered, fluorinated chemicals immediately emerge as the first option. Not in vain, many SHSs are achieved after a chemical modification with fluorocarbons [109,111,178,180]. Menini and Farzaneh [147] coated aluminum alloy coupons with polytetrafluoroethylene (PTFE), using an Al_2_O_3_ underlayer previously produced by anodization to enhance the coating adhesion, as demonstrated by a cross-cut tape test (ranked 5A according to ASTM D 3359-09 [152]). The IAS was measured by the CAT method and an ARF of 2.41 was observed. Likewise, Jafari et al. [189] sputtered PTFE on anodized aluminum alloys, observing an ARF of 3.5, but the icephobicity was lost after several icing/deicing cycles. Even though fluorine-containing substances have been successfully used to prepare icephobic coatings, other alternatives are preferred as they are potentially harmful materials for livestock and the environment.

#### 4.2.2. Non-Fluorinated

Due to the environmental concerns of fluorinated chemicals, other low-surface-energy chemicals have been considered in the design of icephobic materials. The selected low-surface-energy chemicals are often alkoxysilanes, even though it has been demonstrated that they tend to be less effective towards icephobicity under the same conditions [190]. Donadei et al. [118] flame sprayed a low-density polyethylene powder with fully hydrogenated cottonseed oil powder as a lubricating additive to prepare icephobic coatings. The as-prepared coatings were hydrophobic (90° < WCA < 150°) and showed an IAS below 50 kPa (measured with the CAT method) that increased with only four icing/deicing cycles, as repeated cycles caused mechanical damage to the surface. Allahdini et al. [149] prepared an icephobic coating with an alkoxysilane binder and fumed silica nanoparticles embedded in a conventional silicone resin (Silikophen AC 1000) by spray coating, enhancing the WCA of the resin from 79° to 163°. The SHS showed an IAS of 13.3 kPa that increased to 15.4 kPa after 15 icing/deicing cycles. In addition, the coating showed a good adhesion to aluminum, steel, glass and porcelain rating as determined by the cross-cut test (ASTM D 3359-09 [152]). The coating remained superhydrophobic after 30 cycles of tape peeling (3M scotch tape), 400 cm of linear abrasion test (1200 grit sandpaper, 5 kPa) or immersion in various solutions with pH ranging from 2 to 14 for 24 h. 

### 4.3. Icephobic Coatings with Low Shear Modulus

A large modulus mismatch between the ice and the surface to be detached from promotes the ice shedding off, as originally described by Kendall [191] and further extended to shear by Chaudhury and Kim [192]. Taking into account that the shear modulus of ice (obviously affected by temperature and type of ice) is typically in the order of a few GPa [193], soft rubbery materials such as silicones [194] and polyurethanes [195] are the most representative low-shear-modulus icephobic materials. Regarding the durability of the icephobic coatings with a low shear modulus, it should be highlighted that it is intrinsically limited by their own nature, as it relies on the softness of the coating and thus usually leads to poor mechanical properties [196]. 

**Figure 12:**
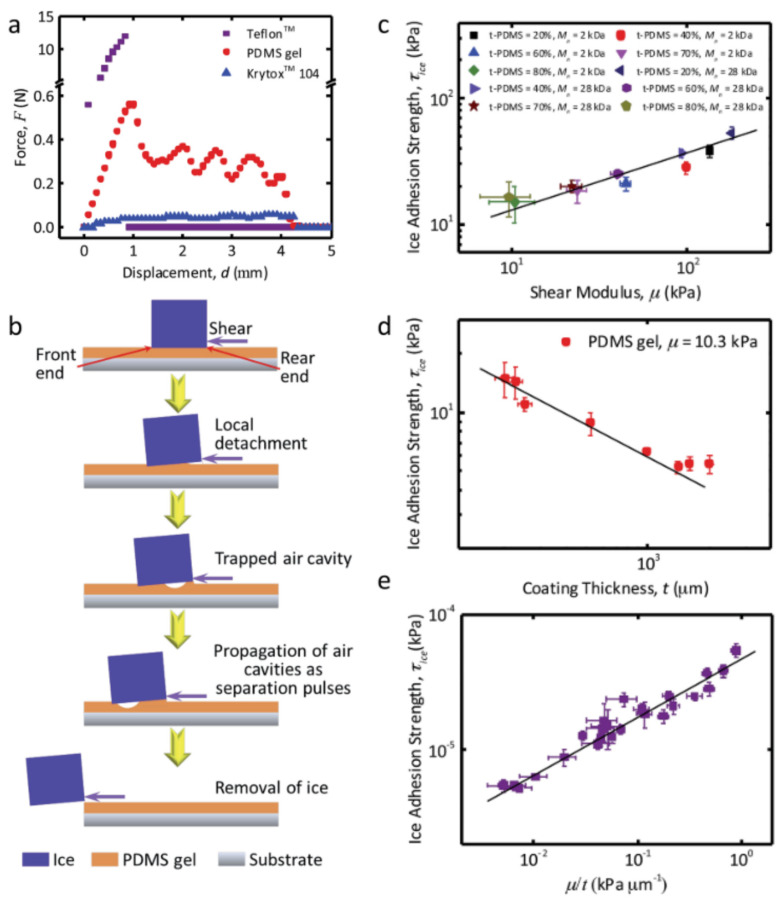
PDMS gels with ultra-low adhesion to ice: (**a**) Force-displacement curves obtained during detachment of ice from the surface of Teflon (rigid hydrophobic material), a PDMS gel (soft hydrophobic material) and Kritox (a liquid lubricant at a shear rate of 0.8 mm/s; (**b**) Schematic illustration of the interfacial cavitation; (**c**) Ice adhesion strength plotted against the shear modulus, (**d**) the coating thickness, and (**e**) the ratio of shear modulus to coating thickness. Reprinted with permission from [112]. Copyright (2016) Royal Society of Chemistry.

**Figure 13:**
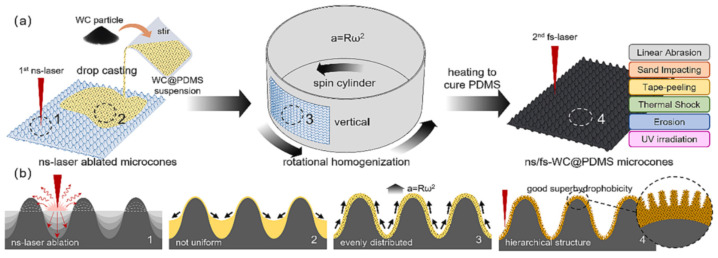
Schematic fabrication of the WC@PDMS coating: (**a**) The process was divided into three stages (ns-laser processing of microcones, rotational homogenization of WC-doped PDMS suspension and secondary fs-laser ablation); (**b**) The evolution of microstructures in Areas 1, 2, 3, 4. Reprinted with permission from [197]. Copyright (2023) Elsevier.

Beemer et al. [112] prepared a series of PDMS gels where the shear modulus was tuned by adding different amounts of a non-reactive trimethyl-terminated PDMS, which acted as a plasticizer by impeding the cross-linking and thus lowering the shear modulus. The relationship between the IAS and the shear modulus of the icephobic surface and the coating thickness was corroborated (Figure 12c–e). In addition, the force-displacement curve during the ice detachment in shear mode (mode II) was reported and compared with the one corresponding with a hard hydrophobic surface such as PTFE (Figure 12a). On top of that, they elucidated the mechanism of separation of ice from PDMS gels for the first time and referred to it as separation pulses (Figure 12b), which is also known as the interfacial cavitation mechanism (Table 4). They also tested the durability of the PDMS gels and observed that the IAS remained unchanged even after 100 icing/deicing tests and showed a slight increase (<2 kPa) after 1000 abrasion cycles (400 grit sandpaper, 6.8 kPa). Tailoring the shear modulus of PDMS gels has allowed researchers to achieve an IAS of 0.15 kPa only [57], which is five orders of magnitude lower than the IAS of aluminum. On the one hand, such an ultra-low IAS allowed the ice to slide off solely under its own weight; on the other hand, the IAS increased with icing/deicing cycles as the surface began to degrade. The PDMS coatings were easily damaged after 20 abrasion cycles (Taber abrasion test, weight of 1100 g). Similarly, Ibáñez-Ibáñez et al. [120] analyzed the mechanical durability of PDMS gels with different elasticity (by modifying the cross-linking agent to the silicone base ratio). They observed that the moderate elastic surface presented a good durability although the IAS increased after wear. Zhou et al. [116] prepared a flexible norbornene-based fluorinated polymer with an IAS below 20 kPa for at least 50 icing/deicing cycles, which was considered as proof of excellent durability. Zhuo et al. [67] prepared a slide-ring cross-linked PDMS coating with an IAS of 13 kPa that did not increase remarkably after 20 icing/deicing cycles and increased only gradually after 800 abrasion cycles (400 grit sandpaper, 1.5 kPa). Recently, Chen et al. [197] incorporated tungsten carbide to increase the durability of a PDMS-based coating against abrasion. Following the scheme shown in Figure 13, they ablated a sheet of aluminum to produce a microcone-patterned surface that was treated with a PDMS solution with dispersed WC powders to obtain an evenly distributed coating that was further ablated to achieve a hierarchical structure. 

**Figure 14:**
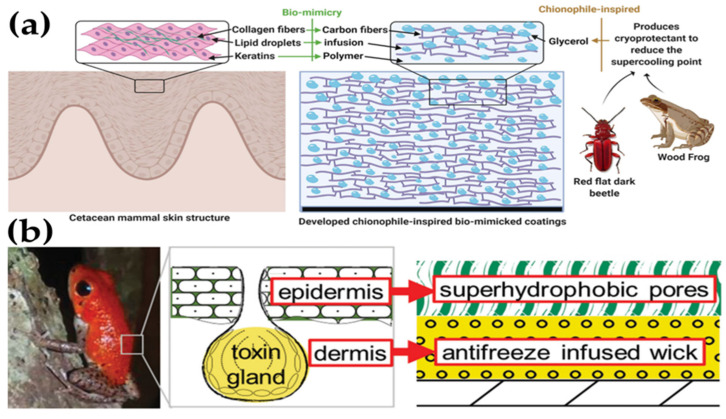
Bioinspired icephobic surfaces. (**a**) Icephobic surface based on the mimicking of the anatomy of a cetacean mammal skin and inspired by the cryoprotective properties of wood frog and red flat bark beetles. Reprinted with permission from [136]. Copyright (2012) American Chemical Society; (**b**) Schematic of anti-icing coating inspired by the functionality and bilayer architecture of a poison dart frog skin, where the porous superhydrophobic epidermis separates the antifreeze-infused dermis from the environment. If water penetrates the surface, the freezing point depressant mixes with water and prevents ice formation and accretion. Reprinted with permission from [221]. Copyright (2015) John Wiley and Sons.

Under the optimal conditions (Figure 14), the 70 μm pitch samples showed superhydrophobicity (WCA and SA of 159.3°and 1.6°, respectively) and an IAS of 6.3 kPa, respectively, which remained stable for the first 20 icing/deicing cycles but increased up to 26 kPa after another set of 20 icing/deicing cycles. The coatings could withstand a linear abrasion test for 240 cm (1000 grit sandpaper, 5.2 kPa), sand impact of 80 g (sand particles falling from 40 cm and impacting on the samples tilted 45°), 200 cycles of a tape-peeling test (tape with an adhesive strength of 710 N/m) and thermal shock (between −40 °C and 200 °C), as demonstrated by a limited enhancement of the WCA. The UV irradiation effect was also evaluated (365 nm, power of 36 W, stand-off distance of 20 cm) and the IAS values remained within the uncertainty of the measurements after 48 h exposure. Following a very different strategy, Wang et al. [135] prepared a mechanically-resistant porous nickel skeleton foam filled with PDMS through impregnation and curing. An IAS of around 6 kPa was measured, which increased to 7 kPa after 90 min of a water–sand impinging test (1 wt.% sand suspension, impinging velocity of 37 m/s, liquid flow rate of 72 mL/min, nozzle to sample distance of 4 cm). Zhuo et al. [198] highlighted that a low modulus does not mean a compulsory low mechanical endurance, as it also depends on the toughness of the coating. They reported a supramolecular silicone elastomer containing octuplet hydrogen bonding, which led to a material with a fracture toughness of 16.43 kJ/m^2^, which is about 46 times higher than that of Sylgard 184 (0.35 kJ/m^2^). This coating presented a stable IAS of around 65 kPa during 35 icing/deicing cycles, and could be healed by annealing at 100 °C for four days after damage impinged through abrasion (400 grit sandpaper, 1.5 kPa) or through a cut with a scalpel. They also prepared this material in a spongy structure by using a salt-based template. The spongy structure further decreased the elastic modulus of the coating and promoted the deformation incompatibility between the ice and the surface resulting in an IAS of 26.7 kPa. This sub-structure promotes the formation of macro crack initiators that facilitate the detachment of ice, as will be discussed in Section 4.5. 

In addition to the use of PDMS, other authors have explored the use of PU-based coatings as they offer superior mechanical strength and durability, toughness or abrasion resistance in comparison with PDMS. The main drawback is that PU-based coatings are rather hydrophilic in nature, so decreasing the polar nature of PU to enhance their icephobicity while retaining the mechanical properties has arisen as an icephobic strategy, mostly with fluorinated compounds [132,195]. As it has been already commented at the beginning of the section, very often icephobic materials can be classified in more than one category. This is the case of the work of Fu et al. [132], who prepared a fluorinated PU-based nanocomposite coating. The icephobic coating had superhydrophobicity and self-healing capability, and it was discussed above in Section 4.1. Both silicone [194] and polyurethane [195] gels have interconnected pores that can host a lubricant (either aqueous or non-aqueous) [157] to prepare a new type of icephobic material, which takes us to the next section.

### 4.4. SLIPS and Organogels as Icephobic Coatings

In a similar way as the lotus leaf inspired the development of SHSs, the Nepenthes pitcher plants inspired the slippery liquid-infused porous surfaces (SLIPS), first reported by the group of Joanna Aizenberg [199]. SLIPS consists of a nano/microstructured porous material which is infused by a lubricant fluid (replacing the air in SHSs) immiscible with the liquid that it is intended to repel, using the original description of Wong et al. [199]. Organogels are semisolids in which an adequate amount of either infused liquid or miscible polymeric chains can induce interfacial slippage at the ice–solid interface by the formation of a lubricating layer. Due to the deep similarities, organogels will also be discussed in this section. Under this premise, both PDMS and PU gels and other polymeric networks have been infused by different oils [57,65,138,196,200], plasticizers [196] and even an aqueous lubricant layer [201] and low IAS values have been observed. Moreover, it has been observed that SLIPS can also act as icephobic materials by lowering the nucleation temperature of supercooled water [202]. Even though the water droplet motion on a SLIPS surface is typically slower compared to an SHS, SLIPS are intrinsically smooth and stable during freezing under high humidity conditions. It is well known that typically the IAS of SLIPS increases rapidly with the number of icing/deicing cycles, mostly due to the lubricant loss and porous texture degradation [65,203]. The lubricant loss from the uttermost surface can usually be restored by the lubricant reservoir of the bulk, but this self-healing capability is usually limited to several icing/deicing cycles [65] and entails environmental concerns.

#### 4.4.1. Enhanced Durability Strategies for SLIPS and Organogels

To enhance the durability of SLIPS, different strategies have been adopted. Vogel et al. [204] improved the stability of SLIPS by a closed-cell architecture made of silica which was impregnated with a fluorinated lubricant. An IAS of 10 kPa was observed and the mechanical stability was addressed by soft methods such as tape-peeling, touching or wiping the surface and then observing the silica scaffold microstructure by SEM. Likewise, Barthwal et al. [65] prepared a durable SLIPS by infiltrating PDMS and low-surface-energy materials on an aluminum substrate nano/microstructured by chemical etching and anodization. An initial IAS of 22 kPa was measured, which increased to 81 kPa after 15 icing/deicing cycles, to 55 kPa after four months’ exposure to the ambient environment, or to 186 kPa after a 100 cm linear abrasion test (1000 grit sandpaper, 200 g load). Rao et al. [205] improved the durability of SLIPS by allowing strong intermolecular forces between the lubricant and the porous surface, as the latter was modified with the same functional group. In addition, the SLIPS they synthetized had a magnetic-thermal and photo-thermal response and so will be discussed further in Section 4.7 (smart icephobic materials). Other authors have utilized the porous nano/microstructure of metal–organic frameworks (MOFs) to immobilize the lubricating layer, obtaining surfaces with an IAS around 10 kPa maintained for tens of icing/deicing cycles [206,207]. As an alternative strategy to strongly attach the lubricant to the polymeric network, Coady et al. [208] utilized UV-cross-linked interpenetrated siloxane polymer networks to enhance SLIPS durability. They observed values of IAS below 10 kPa, but the IAS increased rapidly with the icing/deicing cycles in spite of their efforts. Similarly, Gao et al. [123] prepared functionalized PDMS with polyhedral oligomeric silsesquioxane (POSS) as a cross-linker in which silicone oil was added as a lubricant. They observed IAS values as low as 11.2 kPa that remained below 14 kPa even after 50 icing/deicing cycles or more than 400 cm of a linear abrasion test (400 grit sandpaper, 4.9 kPa). Wang et al. [209] fabricated a durable SLIPS network by pouring a SiO_2_-UHMWPE (ultrahigh molecular weight polyethylene) suspension on the substrate that was subsequently infused with a fluorocarbon, kerosene or silicon. The substrate could withstand severe abrasion (200 grit sandpaper, 2.25 kPa, 1000 cm), 40 cycles of tape-peeling (Scotch-600 adhesive tape, 12 kPa), long term water dripping (100 μL water droplets, height of 30 cm, 90 drops/min at a speed of 2.5 m/s) or severe chemical corrosion (immersing the sample into either hydrochloric acid/sodium hydroxide solution or organic liquids for 24 h). Sarma et al. [117] prepared a durable icephobic organogel based on the quasi-liquid lubrication provided by grafted flexible polymers, which could withstand the heating test (60 °C for 2 h), water impingement (sample was held under a water faucet for 30 min at a volume flow rate of 0.2 L/s) or wiping test with a regular windshield wiper. The IAS was maintained after those durability tests below 20 kPa for 100 icing/deicing cycles. Chen et al. [121] grafted cross-linked hygroscopic polymers inside the micropores of silicon wafer surfaces to prepare a highly durable icephobic coating with a self-lubricating water layer. The IAS remained around 68 kPa as the temperature was lowered until −25 °C, where a sharp increase in the IAS was observed and ascribed to the solidification of the self-lubricating layer and onset of a mechanical interlocking effect between the ice and the textured surface. The surface exhibited an excellent capability of self-healing and abrasion resistance, as it could maintain its IAS around 60 kPa even after 160 cm of linear abrasion (10,000 mesh sandpaper, 12.5 kPa). Dou et al. [201] prepared an anti-icing coating with an aqueous lubricating layer that resulted in a reduced IAS of around 27 kPa that remained almost the same after 30 icing/deicing cycles and without obvious changes from −15 °C to −53 °C when it increased abruptly, as previously observed by Chen et al. [121]. Recently, Wu et al. [210] prepared a SLIPS with improved durability by using a microporous nickel scaffold impregnated with PDMS and different amounts of silicone oil (up to 50 wt.%). The durable SLIPS showed a freezing delay, ice growth rate reduction and an IAS below 16 kPa. The higher the amount of silicone oil, the lower the IAS they observed, stable up to 50 icing/deicing cycles, with the sole exception of the sample with the highest amount of silicone oil. In addition, surface damage was observed after 30 cycles, but it was restored by a self-replenishing mechanism. This self-repair mechanism is inherent with the concept of SLIPS; not in vain, it was already described in the pioneering work of Wong et al. [199] where the first SLIPS was reported. Typically, the lubricant layer or mobile organic chains can migrate from the underlying substrate by wicking into damaged sites. Even though this intrinsic self-healing capability is beneficial, it is usually limited to very few damage/healing cycles by the consumption of the mobile phase, and eventually results in the loss of the ice-repelling capability after some icing/deicing cycles. 

#### 4.4.2. Self-Healing SLIPS and Organogels

In the addition to this lubricant related self-healing capability, the durability of SLIPS can be further enhanced by providing adequate chemical groups in the host phase in such a way that new bonds can be formed after mechanical damage to repair it. Following this approach, Zhuo et al. [211] synthetized an organogel network where the PDMS chains were modified with a Fe-Pyridine complex where the coordination bonds between the ligand and the metal were relatively weaker than the covalent bonds of the PDMS gel. The presence of reversible metal–ligand coordination bonds in combination with the high mobility of the polymeric chains allowed the Fe-Py-PDMS material to fully recover at room temperature in 24–48 h after being cut with a scalpel. They mixed the Fe-Py-PDMS with a commercial PDMS (Sylgard 184) and observed that a Fe-Py-PDMS:Sylgard wt. ratio of 7:1 had a very low IAS, which increased within the first 15 icing/deicing cycles to 12.2 kPa, where it kept steady for at least another 35 cycles. In addition, they cut a cross throughout the coating surface, left the sample self-heal for four days and then tested the IAS of the healed surface. The IAS of the healed surface was 16 kPa, which is slightly higher than the pristine one. More recently, the same group prepared an icephobic organogel in which the self-healing capability was based on hydrogen bonding [212]. The icephobic coating showed low ice adhesion around 50 kPa for 20 icing/deicing cycles and even after a cutting/healing process. 

### 4.5. Icephobic Surfaces by Stress Concentration at the Ice–Surface Interface 

According to fracture mechanics, the shedding of a block of ice from a given surface can be promoted by the stress concentration at the ice–surface interface. The critical strength (IAS) above which the fracture occurs can be estimated using a modification of Equation (9) that results in the following Equation (10) [41,213,214]:

(10) $$\tau_{ice}=\sqrt{\frac{E\gamma }{\pi a\Lambda }}$$

where *E* is the apparent elastic modulus, *γ* is the surface energy, *a* is the crack length and *Λ* is a non-dimensional constant determined by geometric parameters of the interface crack. Within this approach, the icephobic effectiveness of the surface relies on its ability to promote high-stress localized spots at the ice–material interface to induce cracks and consequently minimize the ice adhesion of the material [53]. Chen et al. [215] designed a surface with pits on the surface filled with water or a low freezing point solution covered by an elastic membrane. The purpose of such a combination was to generate swelling force upon phase transformation of the low-freezing-point solution. It was demonstrated that the swelling force was able to produce a film deformation at the ice–solid interface that promotes the shedding of an ice block. Phase change materials (PCMs) are excellent candidates and thus the potential anti-icing capability of encapsulated PCMs embedded coatings has become an active research topic [216]. PCMs produce local shear stress on surfaces due to their volume change (phase transformation) during icing conditions and also release high amounts of latent heat, as they possess a high heat storage capacity [6,217,218]. Given this stimuli-response behavior, they can be considered smart icephobic materials and thus will be discussed later as part of Section 4.7.

#### 4.5.1. Icephobic Materials with Crack Initiators

Zhiliang Zhang’s group introduced the concept of a multiscale crack initiator to promote super-low ice adhesion surfaces [41,214]. They created stiffness inhomogeneities in a PDMS gel by the introduction of hollow sub-structures, reaching an IAS as low as 5.7 kPa. On the other hand, the durability of the materials was not evaluated but it can be inferred that a PDMS gel with patterned hollows will not be mechanically stable enough for many practical applications. They also prepared PDMS foams [122] with submicrometer pores and observed an IAS below 20 kPa after 50 icing/deicing cycles and less than 30 kPa after acid/base/salt/organic corrosion and 1000 abrasion cycles (400 grit sandpaper, 1.5 kPa).

#### 4.5.2. Icephobic Materials with Low Interfacial Toughness

Recently, the group of Kevin Golovin designed buckling elastomer-like anti-icing metallic surfaces (BEAMS) that exhibit an ultra-low IAS (<1 kPa) combined with the mechanical resilience of metals [110]. Alasvand Zarasvand et al. [110] hypothesized that, if ice were adhered to a thin metallic plate, the shedding of the ice could be facilitated by the crack-opening displacements induced by the deflection of the plate during buckling. They compared the IAS of fully confined metals such as steel, aluminum or brass with their BEAMS counterpart and observed in all the cases IAS below 1 kPa for the BEAMS while the IAS of the fully confined metals falls around 500 kPa. A complete set of durability tests were conducted and the superior durability were demonstrated (Figure 8a). Golovin et al. [63] successfully prepared low-interfacial-toughness (LIT) materials for which the force required to detach ice from large areas is low and independent of the interfacial area, above a critical length. They were able to prepare LITs with common plastics such as polyethylene, polypropylene and polystyrene, but this was not achieved with PDMS rubber. They observed that for LIT materials the force per unit width required to debond ice from the surface increases up to a critical length where a plateau is reached, as they operate in the toughness-controlled regime (Table 4). This is manifested by the rapid propagation of an interfacial crack. 

### 4.6. Bioinspired Icephobic Coatings

Nature has become an endless source of inspiration for the development and design of icephobic surfaces [37]. In addition to the well-known inspiration of the nano/microstructure of lotus leaves to design the SHSs [219,220] or the Nepenthes pitcher plants to develop the SLIPS [199], many other biological systems have inspired other icephobic surfaces, such as the skin of the dart frog [221],the red flat bark beetle or the wood frog [136], the shell of the mussels shell [153,222], the nano/microstructure of the butterfly wings [223] or penguin feathers [224,225]. Memon et al. [136] developed a coating mimicking the anatomy of a cetacean mammal skin structure where the collagen fibers are replaced by carbon fibers and the keratins by a polymer. In addition, glycerol was used as a cryoprotectant chemical to reduce the supercooling point, bioinspired by the red flat dark beetle and the wood frog (Figure 14a).

Sun et al. [221] prepared a bilayer architecture structure inspired in the skin of the dart frog (Figure 14b). The bilayer structure was composed of an inner hydrophilic membrane infused with a freezing point depressant and an outer porous SHS. Under dry conditions, the surface exhibited typical superhydrophobic behavior. However, if water penetrated the surface (high-velocity impact drops or condensation frosting), the freezing point depressant mixed with the water preventing ice formation and accretion. This stimuli-responsive behavior reduces the required antifreeze replenishment rate, which enhances the durability of the coating. 

The use of antifreeze proteins (AFPs) and its mimics in icephobic coatings have been considered as well [30], inspired by the antifreeze proteins found mostly in Arctic fish [226,227]. The processing of this type of materials is typically complex and the durability limited. Not in vain, the durability is usually evaluated by measuring the immobilization of the AFP on the substrate [228]. In addition, icephobic coatings based on AFPs are good icephobic materials in terms of freezing delay but might suffer from high IAS once the ice has been formed and accreted, which limits their applications mostly in the food industry, agricultural and cryomedicine [227]. Thus, this type of icephobic material will not be further discussed herein. 

### 4.7. Smart Icephobic Materials

The stimuli-responsive icephobic behavior of smart icephobic materials has attracted significant attention, as their passive/active nature typically enhances the anti-icing/deicing capability [158]. An active deicing system requires an external energy input to trigger the deicing action that usually implies a cost. In contrast, in the realm of smart icephobic coatings, the energy input is commonly provided by nature (e.g., solar light) to trigger the desired response (e.g., heat). Nevertheless, the classification of smart coatings can be very inclusive if the stimuli-response criteria are strictly applied (Table 5), which has been performed here in order to provide a general overview at a glance. If the shedding of an ice block from a surface is desired, the possibility of heating up such a surface is an obvious advantage, so the thermoresponsive smart icephobic materials are key. As soon as a thin layer of water is formed through the melting of the ice at the ice–solid interface, the IAS is weakened and the ice block can be easily removed. Electrothermal coatings can be heated up by the application of an electrical voltage [5,19,29,135,229]. Electrothermal coatings’ Achilles heel relies on the energy consumption, and so the research efforts are mostly focused on reducing it. However, herein we will focus mostly on the smart icephobic systems with the advantage that the stimulus is received by the system at no energy cost (e.g., photothermal systems). 

Electro-/photothermal systems can be applied in combination with passive anti-icing surfaces, where only a moderate IAS (i.e., below 100 kPa) is required [234], so there is no need to use extremely low IAS surfaces which typically suffer from low durability which limits their practical applications. 

#### 4.7.1. Photothermal Icephobic Materials 

As conventional active deicing systems (i.e., electrothermal) typically suffer from low efficiency and high energy requirements, photothermal icephobic materials emerge as a promising alternative for outdoor anti-icing applications [235]. Icephobic photothermal systems absorb solar photons and convert them into thermal energy, by using different photothermal materials such as carbonaceous materials [75,76,131], plasmonic systems [236] or semiconductors [197,230,231], among others [235]. 

Zheng et al. [75] prepared a PDMS-based flexible superhydrophobic photothermal film with Fe powder and candle shot (PFe-PCS) that conferred photothermal activity even under 0.5 sun irradiation (1 sun is equivalent to an irradiation of 1.0 kW/m^2^). The PFe-PCS surface temperature increased about 45 °C and 65 °C within 5 min under 1 and 1.5 sun irradiation, respectively, while the uncoated glass surface temperature increased less than 5–10 °C. The coating also showed a significant freezing delay time (4.7 times of that of the bare substrate). They also tested the photothermal deicing properties by freezing a 200 μL water droplet at −20 °C in the dark. Then, a lamp was used to simulate 1 sun irradiation and the time required to melt the ice was measured, observing that the PFe-PCS surface required 72 s while the coating with only carbon soot required 138 s and with only iron required 200 s. The total freezing time remained between 3 and 4 times that of the bare substrate after immersion in solutions of pH = 1, pH = 12, 2.5 wt.% NaCl for 72 h, 30 min in liquid N_2_, 500 bending cycles, 1600 cm to and fro of linear abrasion test (2000 grit sandpaper, 1.12 kPa), 3 h of water impact test (velocity of 22 cm/s to simulate rainwater impact). In addition, the surface showed self-healing capability, as it recovered its superhydrophobicity after damage with O_2_ plasma (3 min) by resurfacing with a 600 grit sandpaper or solar heating. Wu et al. [232] prepared a superhydrophobic coating containing Fe_3_O_4_ nanoparticles infiltrated in a fluorinated epoxy resin with a freezing time delay of 35 min. The ferric oxide nanoparticles conferred photothermal activity, so the surface temperature at −10 °C could be rapidly raised above 0 °C within 1 min of IR irradiation. In addition, the coating maintained its superhydrophobicity after 400 cycles of peeling test (Scotch tape with adhesion strength of 2600 N/m), 260 cycles of sandpaper abrasion (360-grit sandpaper, 5 kPa) or a sand impact test (sand particles of 355–710 μm in diameter falling from a 30 cm height). Guo et al. [131] incorporated carbon nanofibers onto a polyvinylpyrrolidone-modified PDMS matrix and prepared an icephobic coating with a photothermal effect. They observed a 34-fold increase in the freezing delay time at −15 °C and an IAS stable for at least 30 icing/deicing cycles at about 30 kPa. The photothermal activity was tested under a sunlamp (150 W) at different distances and, at the lower distance, the surface of the photothermal coating increased by about 21.6 °C in 5 min whilst the PDMS control sample temperature increased by 5.9 °C. The durability of the icephobic coating was evaluated by a modified water impinging test to simulate acid rain (pH = 0 HCl solution falling from 6 cm at 1 m/s on the sample tilted 45°) and sand dropping test (sea sand of 100–300 mesh falling from 50 cm on the sample tilted 45°). In both cases, a slight increase in the IAS was observed after 3 L of HCl solution or 30 min of sand abrasion test. Finally, it is worth succinctly mentioning the work of Chen et al. [197] (see Figure 13), who incorporated WC to a PDMS-based coating to enhance its durability as well as the photothermal activity. It was observed that the temperature of the sample rose from room temperature to 63.4 °C after 10 min of sunlight irradiance.

#### 4.7.2. Magnetosensitive Icephobic Materials 

**Figure 15:**
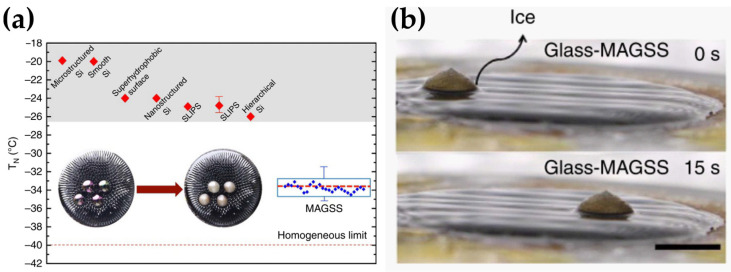
Magnetic slippery surfaces (MAGSS). (**a**) Median nucleation temperature of MAGSS is compared with other icephobic surfaces. (**b**) A water droplet moves over a MAGSS surface tilted 2.5°. Scale bar, 1 mm. Reprinted with permission from [233]. Creative Commons CC BY (2016) Peyman Irajizad et al.

In addition to the typical electrical energy input of the electrothermal systems, the driving force can also be a magnetic field in the so-called magnetosensitive icephobic materials [205,231,233]. Magnetic responsive deicing systems generate heat through Néel or Brownian relaxation and involve several advantages in comparison with electrothermal systems, such as real-time response and lower energy consumption [158]. Rao et al. [205] incorporated Fe_3_O_4_ nanoparticles on icephobic SLIPS with a remarkable icing delay. These coatings had considerable magnetosensitive, photothermal and self-healing properties. They observed that the magnetization saturation values increased with the Fe_3_O_4_ content and the conversion of magnetic energy into thermal energy allowed for an increase in the temperature of 22 °C with an operating power of 450 W. Regarding the photothermal activity, an increase of 20 °C was observed on the surface after 20 min of sunlamp irradiation exposure (75 W). In addition, the matrix of the coating had reversible disulfide bonds that conferred good self-repairing properties. Likewise, Cheng et al. [231] introduced Fe_3_O_4_ nanoparticles in a fluorinated epoxy resin to prepare superhydrophobic coatings with a freezing delay of around 47 min and both photothermal and magnetothermal effects. Using a high frequency induction heater operating at 35 kW, the temperature of the surface increased from 24 °C to 44 °C in 25 s. Regarding the photothermal behavior, the temperature rose from 25 °C to 38 °C in five minutes. Here, it should be highlighted that the photothermal experimental setup was carried out using a sunlamp to simulate a solar light source, so it can be considered a passive system. They also measured the time required to melt an ice layer under a sunlamp and it was around 50% lower for the coating with the highest amount of Fe_3_O_4_ nanoparticles in comparison with the coating with no ferric oxide. In addition to the thermal response to a magnetic field, it has been demonstrated that with the use of ferrofluids, the surface morphology can be strongly modified with the intensity and orientation of an external magnetic field. Irajizad et al. [233] used a ferrofluid along with a magnetic field to develop magnetic slippery surfaces (MAGSS). MAGSS showed an extraordinarily low ice nucleation temperature of −34 °C, much lower than other values reported in the literature, that typically fall above −26 °C (Figure 15a). Regarding the freezing delay time, MAGSS outperforms other surfaces reported in the literature with ice nucleation times around 2–3 orders of magnitude higher. The IAS of MAGSS is ultra-low, stable for 60 icing/deicing cycles at about 2 Pa, which is five orders of magnitude lower than the reported values for SHSs and SLIPS. This extraordinary ultra-low IAS is achieved through a magnetic slippery liquid–liquid interface, which allows the sliding of the ice by a minimal force preventing ice accretion (Figure 15b).

In the presence of a magnetic field, the ferrofluid surface topography is composed of surface waves (Figure 15) that change with the magnetic force and orientation. The MAGSS surface also has self-healing characteristics as it remains unaffected by scratch or shear flows up to a Reynolds number of 10^5^, due to the fluid-like properties of the MAGSS combined with the strong internal cohesion provided by the magnetic field. 

#### 4.7.3. Phase Change Materials

PCMs are characterized by a high heat storage capacity that allows the material to adsorb/release a high amount of heat during a phase change process (melting–solidification transformation) that occurs at a given temperature. To be used in icephobic applications, the melting temperature of the PCM should be near above-zero and the high latent heat values should be accompanied by a marked thermal expansion during solidification. Thus, PCMs can be encapsulated and embedded in polymeric matrices to prepare icephobic coatings [216]. It has been demonstrated that the use of PCMs can reduce the ice accretion [6], the IAS [6,217] and also produce a significant freezing delay [217,218]. Nevertheless, the use of PCMs for icephobic applications is relatively recent (pioneering studies are from one decade ago [6]) and there is still a lack of studies on the durability of these icephobic systems. 

#### 4.7.4. Other Types of Smart Icephobic Materials 

Electromechanical (piezoelectric) materials are of special interest, as they have the ability to produce surface deformations through mechanical pulses in response to an electrical current. They are integrated by a piezoelectric material that vibrates when an electrical field is applied, generating shear stress on the ice–surface interface that can promote the shedding of the ice [23,26]. 

**Figure 16:**
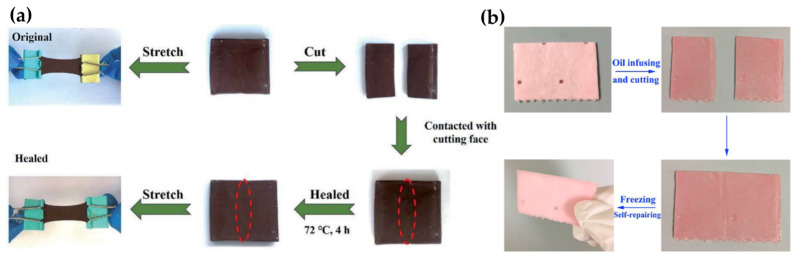
Self-healing icephobic systems. (**a**) SLIPS with self-healing capability based on reversible disulfide bonds formed in 4 h at 72 °C. Reprinted with permission from [205]. Copyright (2019) Royal Society of Chemistry; (**b**) Phase transformable SLIPS with self-healing capability based on the mobility of the liquid lubricant above 3 °C to allow the replenishment of the voids/pores and subsequent freezing to mechanically attach both damaged surfaces by the solid lubricant after phase transition. Reprinted with permission from [213]. Copyright (2019) Elsevier.

In addition, there are other two important types of smart icephobic materials: self-healing and self-lubricating materials, whose interconnected boundaries are sometimes blurred. We have already discussed self-lubricating materials in Section 4.4. SLIPS intrinsically have the capability of lubricant replenishing from the bulk reservoir in response to a lubricant loss [65,121,132,136,137,138,210]. In some cases, self-healing can be achieved by a self-lubricant replenishing, but the self-healing capability is typically lost after several damage/healing cycles [121,132]. On the other hand, if the damage does not imply a lubricant loss, such as extensive oxidation simulated by O_2_ plasma treatment, the self-healing capability can be based on the mobility of the lubricant phase and thus it can last many cycles. In those cases, we can consider that the icephobic coating has self-healing characteristics [169,187,188]. Finally, there are some icephobic coatings in which the self-healing capability relies on the presence of reversible chemical bonds such as hydrogen bonds [198,212], disulfide bonds [205] or metal–ligand coordination bonds [211]. As the self-healing process requires the formation of new chemical bonds, the specific thermodynamics and kinetics of the system impose certain minimum conditions. It was observed that the hydrogen bonds could be restored at room temperature during long periods (up to 96 h) [212] or in 6 h only at 100 °C [198], while the metal–ligand coordination bonds required up to 96 h at room temperature [211]. Rao et al. [205] restored the disulfide bonds in only 4 h at 72 °C and the healed sample was even able to pass a stretching test (Figure 16a). Finally, there are other icephobic materials that can use the phase transition of the lubricant to provide self-repairing capability. Wang et al. [213] prepared phase transformable SLIPS with a peanut oil as the lubricant, with a phase transition temperature of 3 °C. After the infusion of the coating, it was possible to cut it into two pieces and heal them by placing the new surfaces in contact one to another at a temperature above 3 °C, so the liquid lubricant could flow by surface-energy-driven-capillary force to the damaged area and replenish the voids/pores. Then, the material was cooled down below the melting temperature of the lubricant, so the solid lubricant was able to interact with strong intermolecular forces and attach the damaged area with a reasonable mechanical strength (Figure 16). 

### 4.8. Nanocomposite Icephobic Coatings

Nanocomposite coatings are not commonly included in a classification of icephobic materials (Table 3) but, at the same time, nanocomposite coatings can be found in the categories already discussed herein. Since the present work is focused on the durability of icephobic coatings, nanocomposite icephobic coatings will be discussed in their own category due to their importance. If an icephobic coating is intended to withstand for a long period under harsh outdoor subzero conditions with an acceptable icephobic performance, it should be damage tolerant. One option is the use of self-healing materials, which is a very appealing strategy, but if the material is lubricant replenishing dependent, it can be limited by the number of damage/healing cycles [65,121,132,136,137,138,210]. If the self-healing material is based on reversible bonds, the healing process might require a thermal treatment [198,205], or a long healing period [211,212], at which the material is mechanically vulnerable. There is another option that relies on a durable icephobic coating with a bulk nanostructure that is chemically equivalent to the surface, so if the material suffers severe damage (i.e., the outermost surface is destroyed) the new exposed material is similar in texture and functionality. Following this strategy, a mechanically durable system can be selected and modified towards the enhancement of its icephobicity while maintaining its superior mechanical properties. 

#### 4.8.1. Epoxy-Based Nanocomposite Icephobic Coatings

Epoxy systems are well known for their superior mechanical durability and physicochemical inertness, so the modification of an epoxy resin to enhance the icephobicity while maintaining its mechanical stability is an appealing choice towards the preparation of a durable icephobic nanocomposite coating. Epoxy resins have been successfully modified to enhance their icephobicity with different chemicals such as fluoropolymers [66], fluorosilanes [237], non-fluorinated silanes [49], PFTE particles [238] or PDMS-grafted SiO_2_ nanoparticles [106]. 

Wu et al. [106] prepared a silicone-epoxy hybrid resin, with PDMS-grafted SiO_2_ nanoparticles with different sizes. To evaluate the durability of the icephobic nanocomposite coatings, they home-designed a micro-sand blaster (silica particles of 63 μm, sprayed at 200 kPa for 30 s at a distance of 10 cm) and measured the IAS and ice accumulation rate before/after sand erosion. They observed that the sample with a dual size of silica nanoparticles had a lower IAS—about 65 kPa—and that it remained below 100 kPa after the severe sand erosion test. Likewise, the ice accretion rate remained below 15% even after sand erosion. In addition, they conducted a UV-accelerated weathering test (cycles of 4 h of intense UV irradiation at 60 °C followed by 4 h moisture exposure at 50 °C) and observed an increase in the IAS of up to 71 kPa while the ice accretion rate remained below 10%. Lv et al. [66] prepared durable icephobic fluorinated epoxy coatings using maleic anhydride as a cross-linking agent. They measured the IAS after and before the coatings were worn down by 0.1 mm with an 800 mesh sandpaper. The measured IAS was between 64 and 128 kPa, which is not very low but the coating demonstrated a superior durability. Here it should be noted that, for many real applications, the mechanical durability of the icephobic coating might be even more important than the icephobic properties itself. Bai and Zhang [237] prepared an epoxy-based nanocomposite coating with graphene oxide and diatomaceous earth, which was further treated with a fluorosilane to lower the surface energy. The coated aluminum substrate had a significant freezing delay (2760 s vs. 485 s for the bare aluminum substrate) and about one fifth of the ice growth rate of the uncoated substrate. They abraded the coating (400 grit sandpaper, 0.8 kPa, 100 cm) and observed that the freezing delay decreased to 2580 s while the ice growth rate increased slightly in comparison to the as-prepared nanocomposite coating. Some of the present authors prepared an epoxy nanocomposite coating with graphene nanoplatelets grafted with low-surface-energy non-fluorinated silanes. An IAS as low as 9 kPa was observed, well below the natural ice detachment threshold, which is usually considered as 20 kPa [57]. To evaluate the durability of the coating, a severe linear abrasion test was conducted (80 grit sandpaper, 5.88 kPa, 200 cm (x-axis) and 200 cm (y-axis) to and fro). The IAS was increased up to 49 kPa, which is still icephobic. Here, it should be noted that the 80 grit sandpaper is very coarse and that the applied pressure was high enough to severely wear the surface, so this could be comparable to the work of Lv et al. [66], as they wore the nanocomposite coatings down by 0.1 mm. 

#### 4.8.2. Non-Epoxy Based Nanocomposite Icephobic Coatings

In addition to the epoxy-based durable icephobic nanocomposites, other polymeric systems have been studied. Gao et al. [104] prepared a series of copolymer/silica nanocomposites in a cross-linked polymer network. They simulated freezing rain by impinging supercooled water (−18 °C, relative humidity of 40%, poured from a 20 cm height). No ice formation occurred on the optimized copolymer/nanoparticle composite, even after abrasion (28 abrasion cycles with a modified Taber testing using a 300 g load). Shen et al. [133] fabricated PDMS-based nanocomposite coatings with fluorinated silica and observed an excellent water repellency, with a WCA of 155 and a very short contact time (about 10.2 ms) of impact droplets, that still rapidly rebounded off the coating surface at −18 °C. The freezing time was more than 50-fold increased as compared with bare aluminum and the measured IAS was 26 kPa. The durability of the coating was not evaluated against abrasion but it was observed that the IAS remained stable after 30 icing/deicing cycles, 48 h of soaking in HCl (pH = 5.6) or impinging by water jet with HCl (7 mm in diameter hose, pH = 5.6, falling from 5 cm at a speed of 1 m/s).

## 5. Conclusions and Prospects

The durability of an icephobic coating is the main factor slowing down the transfer from the lab to the real applications. It is rarely well addressed as many times the coatings are tested under mild conditions only, or because no icephobic performance is evaluated after the durability test. Here, we have reviewed the different durability methods and conclude that it is key to carefully select the appropriate parameters to ensure the coating will be durable enough for a given application. Often, the best solution for a specific application is not the one with the best icephobic performance but the one with the best balance between the icephobic performance and durability, considering as well other aspects such as cost effectiveness, ease of application, environmental sustainability and even the aesthetics of the coating. Comparisons between different systems might be difficult as there is a need for more standardization of the procedures and parameters in order to enable faster screening and material selection. Even when the same method is used in different studies, important parameters can vary from one to another, which hinders a direct comparison. Currently, these include key parameters such as the subzero temperature in the determination of the IAS or the sandpaper coarseness and pressure in an abrasiveness test.

The paper also reviews the strategies to enhance the durability of the different types of icephobic coatings in order to broaden their potential applications. Traditional low-surface-energy approaches such as the use of SHSs or lubricated surfaces have been extensively investigated. An excellent icephobic performance has been already achieved (e.g., low IAS), so the efforts are now mostly focused on enhancing their durability as they typically suffer from mechanical weakness and lubricant depletion, respectively. There are other paths towards icephobicity, such as icephobicity through interfacial cavitation, which can be addressed by the use of low-shear-modulus materials; however, the low mechanical resilience limits their applications. Following the same path, there are other current approaches such as the use of PCMs or surface crack initiators, but more research is required in order to explore their potential and durability limits. On the other hand, there are recent studies focusing on the achievement of highly durable icephobic surfaces through materials with low interfacial toughness, which is very promising. The use of smart icephobic materials, mostly with PCMs or photothermal materials, have a lot of potential since they can activate their icephobic nature when required with no external energy requirement. The use of icephobic materials with self-healing capability is an appealing strategy to deal with harsh conditions, but their practical applications are mostly limited to those environments where the conditions to heal might naturally occur (e.g., self-healing at low temperature, achieved under sunlight).

Finally, when durability is crucial under harsh conditions, the use mechanically stable nanocomposite icephobic coatings is a promising option. In such a case, the properties can be tuned to behave as durable low-interfacial-toughness materials and this even allows us to integrate other desirable properties in the development of advanced multifunctional materials, where active and passive icephobic approaches can be combined to meet the requirements of the most demanding icephobic conditions. 

## Figures and Tables

**Table 1:** Different types of solid water formed by atmospheric icing and their main characteristics to consider from an icephobic point of view, such as density or typical adhesion.

Type of Solid Water	Density	Main Characteristics	Typical Adhesion *
Frost	Low	Ice with sparse dendritic crystal structures formed when water vapor solidifies directly (desublimation) on a cold solid surface. It often occurs during low winds.	Frost adhesion may be strong
Snow	Low to medium	A mixture of small ice crystals formed directly from water vapor (desublimation) in the air. Snow is dry at air temperatures below −1 or −2 °C, but at temperatures closer to the freezing point, a thin layer of water covers the ice crystals, in the so-called wet snow. In the absence of impurities, it shows a white color.	Wet snow presents low adhesion when deposited on a solid surface but it becomes strong when frozen. Dry snow can accumulate in offshore platforms and vessels becoming a hazard to personnel and material.
Rime	Low to medium	White ice with needles and flakes. It is formed when supercooled droplets (5–70 μm) impact on a surface below 0 °C and freeze very fast. Accretion depends on the amount of water droplets in the air, the droplet size, the air temperature, the wind speed, the duration of the event, etc. Soft rime: If the water content of the air is low and the size of the water droplets is small. Soft rime presents low density. Hard rime: If the water content of the air is high and the droplets are bigger. Hard rime presents higher density.	Soft rime presents a low density and little adhesion. Hard rime presents a higher density and it is more difficult to remove.
Glaze	High	Clear, dense and hard ice. It is formed when big supercooled droplets (70 μm to even a few millimeters) impact on a surface below 0 °C. Those big droplets do not freeze immediately upon impact but run on the surface before completely freezing.	Strong
Ice	High	A brittle frozen state of water. It can be transparent or more or less opaque depending on the presence of impurities or pockets of air. It can be formed by freezing rain (rain falls on a surface below 0 °C) or by the presence of water on a surface that cools down below 0 °C.	Very strong

* Typical adhesion is referred to a non-icephobic material such as bare steel or aluminum.

**Table 2:** Different mechanical methods to assess the durability of icephobic surfaces.

Type of Method	Standard	Main Characteristics
Icing/deicing cycles	N/A	The IAS is determined over the same icephobic surface for a high number of times, usually with the peak force method (Figure 1a). If the IAS value is stable with the number of icing/deicing cycles, the durability of the icephobic surface is acceptable. It is a very simple procedure that does not require any specific sample preparation. An icephobic surface that is degraded after several icing/deicing cycles is very weak. A sample that is not damaged has not demonstrated a superior level of resistance.
Sandpaper abrasion test	N/A	The grit of the sandpaper, the applied pressure and the total distance are freely chosen, which allows us to design the test as desired but hinders direct comparisons between coatings of different research groups.
Taber abrasion test	ASTM D4060-95 [124]	The sample is mounted on a rotary platform and abraded against commercially available abrading wheels. The platform rotating speed can be adjusted to 60 or 72 rpm and one full rotation is considered a cycle. In addition to the IAS or WCA, the wear index, weight loss or thickness reduction can be calculated every certain cycle.
Sand impact test	N/A	The icephobic coating is placed a certain distance below a container, from where sand falls down and impacts the coating surface, typically at an angle of 45° or 90°. There are high pressure variations, like the use of sand blasters. Distance, angle and particle size are key parameters.
Water impact test	N/A	The icephobic coating is placed a certain distance below a container, from where sand falls down and impacts the coating surface, typically at an angle of 45° or 90°. There are variations where high pressure water jets are used. Distance, angle and droplet size are key parameters.
Tape adhesion (peeling test)	ASTM D3359-09 [152]	Conventional adhesive tape is placed and pressed against the icephobic surface and then peeled off. This cycle is repeated as many times as desired. This method is very simple, cheap, and do not require any specific sample preparation. The main drawback of this type of test relies on its softness. A surface that shows degradation after the tape adhesion test cannot be considered mechanically stable enough for most applications.
Nano indentation	ISO 14577-1:2016 [139]	A diamond tip indenter is pressed against the surface and the hardness and elastic modulus can be obtained and compared with any other material, as it is very extended. This method is relatively simple, not expensive and does not require any specific sample preparation. From an icephobic engineering perspective, this method provides useful information about the mechanical properties of the coating but no information about the icephobic behavior after mechanical damage.
Pencil scratch test	ISO 15184:2020 [143]	The icephobic coating is scratched with pencils of different hardness and the response of the coating allows us to evaluate the hardness of the coating. No information about the icephobic behavior after mechanical damage.
Cross-cut test	ISO 2409:2013 [146] ASTM D3359-09 [152]	The icephobic coating is X-shaped cross-cut with blades through the coating until the substrate and an adhesive tape is placed and pressed over the damaged. The tape is pulled-off and the coating/substrate adhesion is evaluated from the spalled area. No information about the icephobic behavior after mechanical damage.
Dolly pull-off test	ISO 4624:2016 [151]	A pull stub (dolly) is adhered to the coating using very strong glue. Once cured, the dolly is pulled in mode I and, if an adhesive failure occurs at the coating/substrate interface, its strength can be calculated. Otherwise, it can be affirmed that it is greater than the failure value. From an icephobic engineering perspective, this method provides useful information about the adhesion of the coating to the substrate but provides no information about the icephobic behavior after mechanical damage.

**Table 3:** Different classifications of icephobic materials that can be found in the literature according to the icephobic strategy and/or microstructure.

Authors	Classification
Dhyani et al. [60]	(a) Deicing: surfaces that facilitate the detachment of accreted ice (ice shedding): low surface energy (1), lubricants (2), low modulus (3), mobile polymer chains (4), stress concentration (5) and low interfacial toughness (6). (b) Anti-icing: surfaces that delay the accretion of ice: macro-textured surfaces (1), superhydrophobic surfaces (2), nanoroughness effects (3), lubrication (4), phase change materials (5) and charged and amphiphilic surfaces (6). (c) Snow repellency: surfaces that resist snow accretion.
Liu et al. [30]	(a) Passive anti-icing to prevent freezing beforehand: Superhydrophobic surfaces (1), antifreeze proteins and its mimics (2), ionic polymer surfaces (3) and nanostructured antifrost surfaces (4).
	(b) Active deicing to remove ice after formation: lubricant impregnated surfaces (1) and soft fracture mechanism elastomers (2).
	(c) Multifunctional material with both anti-icing and deicing capability: passive anti-icing compounding with thermally ice melting (1) and passive anti-icing compounding with low ice adhesion strength (3).
Kreder et al. [162]	(a) Dry: smooth and textured surfaces. Dry and smooth icephobic surfaces can be observed in self-assembled monolayers (1) and bulk coatings (2), whereas a textured surface can be also classified as microtextured (3) or nanotextured (4).
	(b) Wet (and thus smooth) icephobic surfaces can be microstructured (1), nanotextured (2), infused polymer (3) and hydrated (4) microstructures.
Lui et al. [157]	(a) Low-surface-energy coatings: fluoride-containing polymer coatings (1), silicon-containing polymer coatings (2) and fluorosilicone copolymer coatings (3). (b) Liquid-infused slippery surfaces: oil lubricated coatings (1) and aqueous lubricated coatings (2).
Yeong et al. [137]	(a) Low-surface-energy polymers/lubricant materials that constitute hydrophobic (1) and superhydrophobic surfaces (2). (b) Lubricated micro/nanotextured surfaces infused with hydrophobic (1) and hydrophilic lubricants (2). (c) Fluorinated silicone rubbers and copolymers. (d) Lubricant-infused elastomers.
He at al. [163]	(a) Smooth surfaces. (b) Textured surfaces. (c) Slippery surfaces. (d) Sub-surface textured surfaces.

**Table 4:** Different icephobic mechanisms and the governing equation to calculate the adhesion strength. The corresponding engineering and test parameters are also shown. Adapted with permission from Dhyani et al. [60]. Copyright (2022) Elsevier.

Mechanism	Governing Equation	Engineering Parameters	Test Parameters
Low surface energy	$F_{ice}\propto \;L_{ice}\gamma_{LV}\left(1+cos\theta_{rec}\right)$	Receding contact angle, $\theta_{rec}$	Surface tension, $\gamma_{LV}$ Length of ice, $L_{ice}$
Interfacial cavitation	$F_{ice}=1.3\;(\frac{L_{ice}}{l})\sqrt{\frac{W_{a}G}{t}}$	Shear modulus, G	Height of probe, l Length of ice, $L_{ice}$
		Work of adhesion, $W_{a}$ Coating thickness, t	
Lubrication	$F_{ice}=L_{ice}\;\eta u{\left[z\;+(\frac{\eta }{\alpha_{i}})+(\frac{\eta }{\alpha_{s}})\right]}^{-1}$	Lubricant viscosity, η Lubricant thickness, z Surface-lubricant CoF *, $\alpha_{s}$	Removal velocity, u Length of ice, $L_{ice}$ Ice-lubricant CoF *, $\alpha_{i}$
Low interfacial toughness	$F_{ice}=\sqrt{2\Gamma E_{ice}h}$ when (*L* > *L_c_*)	Interfacial toughness, Γ	Modulus of ice, $E_{ice}$
			Height of ice, h

* CoF = coefficient of friction, in units of Pa·s/m.

**Table 5:** Different types of smart icephobic coatings.

Type of Smart Icephobic Coating	Stimuli	Response	Reference
Thermoresponsive (a) Electrosensitive (b) Photothermal (c) Magnetosensitive			
	Electrical current	Heat (Joule heating)	[5,19,29,135,229]
	UV/Vis/NIR light Magnetic field	Heat Heat, Displacement ^1^	[75,76,131,197,230,231,232] [205,231,233]
Phase Change Materials	Heat	Heat storage, Displacement ^1^	[6,217,218]
Electromechanical (Piezoelectric)	Electric field	Mechanical pulses	[26]
Self-lubricating	Loss of lubricant	Lubricant self-replenishing	[65,121,132,136,137,138,210]
Self-healing	Mechanical, thermal, O_2_ plasma treatment or other type of damage	Recovery of the original structure ^2^	[169,187,188,198,205,211,212,213]

^1^ Displacement refers to a change in the microtopology of the icephobic surface. ^2^ Very often, the recovery requires a thermal treatment.

## Data Availability

Not applicable.
